# Loneliness in the Digital World: protocol for a co-produced ecological momentary assessment study in adolescents

**DOI:** 10.1136/bmjopen-2024-087374

**Published:** 2024-06-06

**Authors:** Zhuoni Xiao, Sarah Robertson, Emily Long, Robin Flaig, Liz Kirby, Liana Romaniuk, Aja Murray, Heather Whalley

**Affiliations:** 1 Centre for Genomic and Experimental Medicine, University of Edinburgh, Edinburgh, UK; 2 School of Health and Wellbeing, University of Glasgow, Glasgow, UK; 3 Centre for Medical Informatics, University of Edinburgh, Edinburgh, UK; 4 Centre for Clinical Brain Sciences, University of Edinburgh, Edinburgh, UK; 5 Department of Psychology, University of Edinburgh, Edinburgh, UK

**Keywords:** child & adolescent psychiatry, psychometrics, mental health

## Abstract

**Introduction:**

Loneliness has been identified as an important public health issue, peaking during adolescence. Previous research has suggested that social interaction is a key factor in loneliness, and positive social interaction can act as a protective factor against loneliness. However, it is unclear whether there are differing impacts of in-person and online social interaction on adolescents’ loneliness and mental health. Ecological Momentary Assessment (EMA) designs are ideally suited for better understanding these associations.

**Method and analysis:**

In the ‘Loneliness in the Digital World’ study, we will use a co-developed EMA design to capture daily social interactions, loneliness and mental health such as positive and negative emotions, depression and anxiety in approximately 200 adolescents aged 12–15 years. We will combine this with comprehensive information gathered from online surveys. Analysing the data using techniques such as dynamic structural equation modelling, we will examine, among other research questions, the associations between online and in-person social interaction and feelings of loneliness. The results can help inform interventions to support adolescents with high levels of loneliness and poor mental health.

**Ethics and dissemination:**

We received the ethics approval for the data collection from The Academic and Clinical Central Office for Research and Development, followed by the College of Medicine and Veterinary Medicine Ethics panel at University of Edinburgh, and finally reviewed by East of Scotland Research Ethics Service. The results will be disseminated through journal publications, conferences and seminar presentations and to relevant stakeholders such as teachers.

**STRENGTHENS AND LIMITATIONS OF THIS STUDY**

- This study will use an Ecological Momentary Assessment (EMA) design to collect near real-time data, capturing young people’s social interaction experiences, loneliness and moods in their natural environments which minimises recall bias and provides a more accurate reflection of daily life.
- This study used a co-production method involving formulating research questions, determining measurements, establishing an acceptable frequency of prompts for young people, creating appealing design of the app prompts and deciding the optimal timing for prompt delivery, providing young people insight into the project.
- One potential limitation is that young people who agree to engage in EMA study may differ systematically from those who decline, including due to our instruction to consider not taking part if the topic is likely to trigger negative reactions.

## Introduction

Loneliness has significant long-term consequences for physical and mental health.^1^ Higher levels of loneliness predict physical health conditions, poorer mental health, more frequent sleep disturbances as well as greater cognitive difficulties later in life.^2 3^ Loneliness is especially salient for young people as they strive for independence and increasingly prioritise peer over familial relationships.^4^ Previous research has suggested that social interaction is a key factor in loneliness.^5^ During adolescence young people become particularly sensitive to social rejection,^6 7^ placing them at a higher risk of loneliness.^8^ The COVID-19 pandemic has further exacerbated this issue, with increased loneliness observed among younger ages since 2020.^9 10^

Adolescence is a critical period that encompasses puberty, significant brain development^6^ and an increased vulnerability for internalising problems such as social anxiety.^11^ A previous meta-analysis conducted by Solmi *et al* 12 found that across 192 studies the peak age of onset of any mental disorders was age 14.5 years. However, many researchers focus on participants over 16 years, who are easier to recruit and consent. Hence, it is important to thoroughly investigate the understudied younger adolescent population. In this study, we will leverage our established connection with Generation Scotland (GS) to facilitate the recruitment of participants from this less-explored demographic. This strategic collaboration enables us to gain valuable insights into the loneliness and mental health of age group that has been previously overlooked relative to other age groups in this area of research.

An important consideration in studying loneliness is that young people are spending more time engaging with electronic devices and online interaction compared with previous generations where social interaction were more ‘in-person’. Evidence on whether the amount of time young people spend online affects mental health is mixed. Indeed, there may be both risks and benefits.^13^ Some studies have suggested that the increase in social media use is linked to increase in loneliness and mental illness^14^; however, others have suggested no meaningful associations.^15^ This conflicting literature is likely due to limited understanding of how young people interact online, as well as limitations in the way that these interactions are measured. The impact of the digital world on young people’s mental health remains unclear, necessitating nuanced research on online social interactions.^16^

Smartphone and social media usage are pervasive among adolescents, comprising the majority of their total screen time.^17^ In the UK, a substantial percentage (98%) of adolescents aged 12–15 years have smartphones, and it is unsurprising that these devices were their most-used devices for accessing the internet (96%). Additionally, 93% of adolescents actively engage in social media, with WhatsApp being the most-used app among those aged 12–15 years (80%), followed by Snapchat (62%), Instagram (46%) and TikTok (44%).^18^ This is consistent with trends observed in other Western populations.^19^ Samples in the USA and the UK (2021–2022) estimate that the time adolescents spend on smartphones and social media ranges between 1.5 hours to 8.5 hours per day,^20^ with most adolescents spending between 1 and 3 hours per day.^18^ Social media platforms provide nearly constant opportunities for individuals to connect and interact with others, irrespective of the time of day or geographical location.^21^ Nonetheless, it remains unclear whether online and offline social interaction have distinct effects on the levels of loneliness and mental health among adolescents.

The high levels of engagement of adolescents with digital technology can also be leveraged to gather high temporal resolution, ecologically valid research data to begin to address these questions. EMA has gained significant interest since the 2000s, with a notable increase in studies published annually.^22^ Encouragingly, qualitative data suggest that young people find EMA easy to engage with and report that it enhances their understanding of their own behaviours, feelings and experiences.^23–26^ Furthermore, a comprehensive review by Wen *et al* 27 examining compliance to EMA protocols in adolescents and children indicated that, across 42 studies, the weighted compliance rate stood at 78.3%. Moreover, there was no significant difference in compliance rates between studies involving clinical (76.9%) and non-clinical (79.2%) populations. Notably, among non-clinical studies, those prompting participants 2–3 times daily demonstrated a higher average compliance rate (91.7%) compared with studies with more frequent prompts (4–5 times: 77.4%; 6+ times: 75%). The reported compliance rates remained consistent across different durations of the EMA period in both clinical and non-clinical settings. Therefore, EMA holds promise as a method for studying young people’s mental health, particularly in relation to their online world.

A crucial aspect of the study will involve developing best practice guidance on conducting Ecological Momentary Assessment (EMA) in collaboration with the Young Person’s Advisory Group (YPAG). YPAGs, conceptualised to enhance the acceptability and feasibility of research studies, play a crucial role in considering participants’ perspectives and amplifying the relevance and impact of findings.^28^ The increasing prioritisation of young people’s voice, underscored by the UN Convention on the Rights of the Child, is now integrated into research guidance.^29^ There is a growing emphasis on involving young people as ‘co-actors’ in health research. Engaging in research brings various benefits for young people^30^ as well, including meaningful impact, the opportunity to be part of a supportive community, chances for learning and growth and increased opportunities overall. Consequently, involving YPAGs can be considered imperative for maximising the relevance and benefits of a research project in the realm of health research.

This study will explore how various types of social interaction, both positive and negative, impact state loneliness (ie, a more temporary and situational experience of loneliness, which may be influenced by specific events or circumstances^31^ and mental health in adolescents. We will use an EMA design to assess offline/online social acceptance and rejection in young people and examine how different types of social interaction affect state loneliness, emotions and broader well-being.

Core research questions are as follows:

- Does online social interaction and/or in-person social interaction predict later loneliness and mental health?
- Do online social interaction and in-person social interaction have the same associations to loneliness and mental health?
- Are there reciprocal relations among social interaction, loneliness and mental health?
- Does the time of the day moderate these links?

The latter research question was suggested by the YPAG.

Exploratory analyses can be used to answer a range of additional questions such as exploration of moderation of the link between types of social interaction and loneliness by various other traits of young people. We will also develop best-practice standards for EMA research in young people, as well as validate the psychometric properties of EMA measurement tools.

## Method and analysis

### Procedure

There are two phases in the current project—phase I: YPAG and phase II: full-scale study of 200 adolescents. Phase I is focused on conducting patient and public involvement and engagement work with the YPAG to co-produce a detailed protocol for the main EMA study. In phase II, we will launch the full EMA study, based on consultation with the YPAG.

### Participants

Participants are expected to include approximately 200 adolescents aged 12–15 years from Scotland. Participants who take part in the current project will be sampled from the GS study.^32^ GS is the largest family health study in Scotland and follows the health and well-being of multiple generations of families over time. Over 32 000 volunteers have already joined the study and helped research into cancer, diabetes, mental health and other conditions.^32^ The current study is a new study embedded within GS. When young people join GS, their parents or guardians have confirmed that they believe their young person has capacity to understand what is required to enrol in this study, and that this may involve follow-up studies approved by GS (see online supplemental material A, figures S1 and S2 for pathway for joining GS in online supplemental materialssupplementary material A). The data gathered will be linked to participants’ data from the main GS study. Participants will be offered a £20 e-voucher as compensation dependent on their response rates, as determined by the YPAG consultation processes (see online supplemental material A, figures S3 and S4 for study flow).

10.1136/bmjopen-2024-087374.supp1 Supplementary data

**Figure 1:**
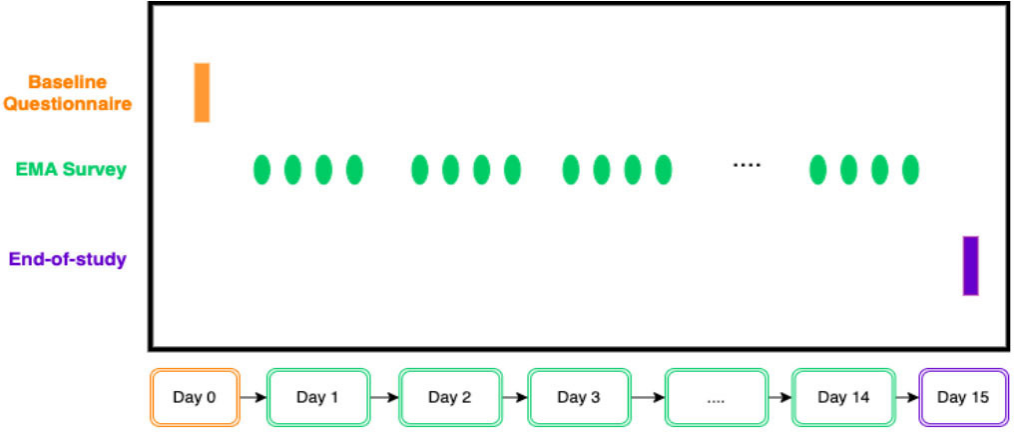
Summary of data collection schedule. EMA, Ecological Momentary Assessment.

### Inclusion and exclusion criteria

Inclusion criteria for the young people will be: (1) aged 12–15 years; (2) resident in Scotland; (3) fluent in English; (4) member of GS; (5) able and willing to install LifeData (EMA data collection) app on their phone; if young people do not have access to a smartphone, our research team will provide one; (6) ability to provide informed consent, and ability to independently complete online surveys and EMA protocols.

Since this study has the goal of encompassing a diverse population with various backgrounds, we have attempted to remove barriers to participation so that we can understand the broader impacts of social interaction on feelings of loneliness. Therefore, there will be no explicit exclusion criteria applied. However, given that some of the questions within the study (included aspects of social interaction, peer relations and mood-related issues) have a small risk of triggering mental health crises, we highlight in the participant informant that young people who have experienced or are currently experiencing negative social interactions should carefully consider whether they wish to participate in the study.

### Patient and public involvement and engagement

Ten young people aged 12–15 years from diverse backgrounds and locations across Scotland were recruited via different platforms. The opportunity was shared via Young Scot and YouthLink e-briefings, LGBT Youth Scotland Discord channel and existing youth work partners, including #iwill Scotland, RespectMe, Passion4Fusion, Glasgow Disability Alliance, Glasgow Young Movers, Glasgow Youth Work Network, Intergenerations Working Together, See Me Scotland and Salvesen Mindroom Centre. The School Health and Well-being Improvement Research Network (SHINE) promoted the opportunity to schools via their newsletter and we delivered a presentation at the SHINE annual conference. In addition, GS promoted the YPAG through posters which were shared on social media platforms including those most popular with young people, such as TikTok and Instagram. Six YPAG meetings were held, and major changes were implemented based on their input to the protocol. For instance, YPAGs suggested that in-person social interactions are more salient than online social interaction; therefore, instead of asking the young people to recall their most impactful social interaction in the past hour, we now randomly ask them to recall their most impactful in-person or online social interaction in the past hour. Additionally, changes were made based on their input, such as reducing the length of the surveys, suggesting the time for each prompt, adding a progress bar for EMA surveys, incorporating a ‘not applicable’ option for all items and embedding more fun and educational elements into the study. These elements include providing cartoon characters for each introduction, offering ‘Fun Images’ and ‘Brain Facts’ at the end of each completed EMA survey. As per the recommendations by Sellars *et al* 33 regarding consistent reporting of YPAG involvement, YPAG characteristics, frequency of engagement, methods and extent of involvement, parental engagement and recognition of involvement are presented in online supplemental material A, table S1.

### Data collection

Data collection will begin in April 2024, and will be completed in 6 months. Figure 1 summarises the study flow. Informed consent will be collected from the young people online, and then they will be invited to complete an online baseline survey.

On completion of the online baseline survey, participants will initiate the EMA surveys. Once researchers confirmed they are members of GS, participants will be provided with a set of instructions for downloading the app, and offered on-boarding support via email, text or call. Participants will use their own smartphone where possible; however, to prevent selection biases and encourage inclusivity, study-provided smartphones will be made accessible. An email helpline and phone number will also be available for participants to receive help on any issues they encounter.

EMA data collection will take place over a 2-week period and involve four prompts per day at 08:00–09:00 hours, 12:30–13:30 hours, 17:30–18:30 hours and 22:00–23:00 hours. All of these will be administered via a smartphone application provided by LifeData. All measures will be designed to take <1 min as lengthy questionnaires are linked to heightened burden and decreased compliance.^34^ Consistent with this, our YPAG emphasised the importance of minimising burden.

**Figure 2:**
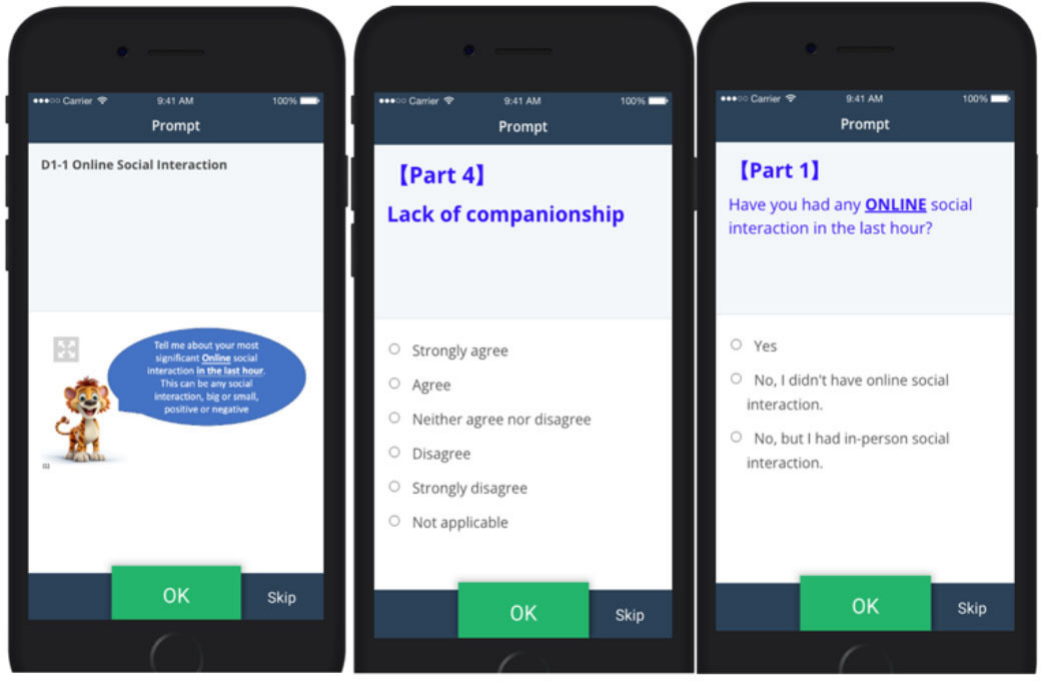
Screenshots of Ecological Momentary Assessment protocol on phone.

At the end of the 2-week EMA period, participants will be offered an e-voucher worth up to £20, with the exact amount to be dependent on their response rates (£5 for completion of baseline questionnaire and different amounts for EMA protocol depend on completion rate—£15 for 70% completion rate; £10 for 50% completion rate; £5 for 30% completion rate; £2 for under 30% completion rate). They will also receive a participation certificate. EMA surveys are provided in the online supplemental material B. Figure 2 presents the screenshots of the EMA protocol on phone.

10.1136/bmjopen-2024-087374.supp2 Supplementary data

### Measures

The baseline questionnaire will be collected online via Qualtrics, and the EMA survey will be collected via the LifeData app. The measures that young people will complete over the course of the study are presented in online supplemental table S2.

### Statistical procedure

Data will be analysed using Dynamic Structural Equation Modelling (DSEM).^35–37^ DSEM combines multilevel structural equation, and time-series modelling, which allows an examination of individual differences in within-person dynamics such as the connections between social interaction and emotions. We will use a multilevel CFA model to examine the psychometric properties of a three-item loneliness measurement. Then, we will use DSEM with lag-1 autoregressive and cross-lagged effects to examine the impacts of social rejection/acceptance on itself over time, and loneliness or emotions at a later time. We will also use mediation within DSEM to explore whether state emotions mediate the associations between social interaction and loneliness or mental well-being. We will be using time-varying effects DSEM to investigate whether time of the day moderate these associations. These models will be implemented in the latest available versions of Mplus and R.

### Sample size justification

We will use a sample size of n=200, adolescents aged 12–15 years. Power analyses are based on a Monte Carlo approach, setting a threshold of α=0.05 and 1−β=0.80. Using this approach, the sample size necessary to detect significant effects in key parameters (eg, moderation of a cross-lagged within-person effect) based on realistic parameter estimates was explored. These realistic parameter estimates were taken from DSEM models fit to past similar EMA data by Murray *et al*.^38^ We determined that an n=100 would provide sufficient power. Therefore, a sample size of n=200 would provide a strong buffer against attrition or missingness, as well as allow us to examine sub-groups such as by gender.

## Ethics and dissemination

We received the ethics approval for the data collection from The Academic and Clinical Central Office for Research and Development, followed by the College of Medicine and Veterinary Medicine Ethics panel at University of Edinburgh, and finally reviewed by East of Scotland Research Ethics Service. The results will be disseminated through journal publication, conferences and seminar presentations.

## Discussion

The current protocol describes the design for a joint traditional and EMA survey study of the daily life social interactions and their impacts of adolescents’ loneliness and mental well-being. We will build capacity and guidance on conducting robust, youth-led research on adolescent mental health, through engagement and methodological innovation. Collaborating with young people aged 12–15 years, we will help contribute to best-practice standards for EMA research, psychometrically validated measurement tools and apply cutting-edge analysis methods for these complex data. We will gather information on whether different types of social interactions have the same impacts on loneliness and mental well-being, whether there are gender differences in these associations, whether social rejection affects subsequent emotions and loneliness and whether current social acceptance would help recovery from previous social rejection by reducing loneliness or improving mental well-being. Our findings can therefore inform better understanding of the impacts of social interaction on loneliness and illuminate poorly understood areas of adolescent mental health with implications for policy and clinical practice. For example, the WHO Commission on Social Connection (2024–2026) aims to see loneliness be recognised and resourced as a global public health priority. Our research could provide preliminary findings for developing solutions for loneliness, as well as valid measurements for assessing loneliness as it plays out in real-time. Our findings can help inform, for example, prescribe culture (eg, better peer relations or better online social behaviours to improve mental health and well-being).

Previous interventions for loneliness focus on different aspects. For instance, social skills,^39–42^ social interaction,^43–45^ emotional skills,^41^ enhanced social support^46–49^ or psychological interventions.^46 50^ However, most interventions are targeted at adults, older adults or patient groups, rather than tailored for the needs of adolescents. Findings from the current project could inform interventions tailored for adolescents and that reflect their social interaction habits such as promoting healthy online behaviours.

Our methodology has the potential to accelerate advancements in the utilisation of EMA technique within adolescent populations in school setting. Through our research, we aim to investigate the impact of prompt timing on response rates, specifically exploring whether prompts during school hours versus after-school hours influence response rates. By providing a template for such research (which we will make openly available in the form of openly accessible study materials, including measures and analysis code), and systematically recording challenges, solutions and advantages, this study can foster broader adoption of EMA methodologies.

### Limitations

While extensive research has explored optimal design features for EMA in general,^34 51^ the relative novelty of the approach for measuring social interactions in adolescents means there is limited past research to guide key methodological decisions such as measure selection, development or validation, sampling frequency and schedules, reimbursement and strategies to promote engagement.^52^ There may also be difficulties in gathering information during school hours meaning that we may have high levels of missingness during the day. Finally, the sample will be drawn from the GS study. While this has the benefit of providing comprehensive measures to link to the EMA data to, the sample may be selective in the sense that they have demonstrated a motivation to engage in health research.

## Supplementary Material

Reviewer comments

Author's manuscript
