# Supplementary material for: Loneliness in the Digital World: protocol for a co-produced ecological momentary assessment study in adolescents
Source: BMJ Open. 2024 Jun 6;14(6):e087374. doi: 10.1136/bmjopen-2024-087374 (PMC11163606; doi:10.1136/bmjopen-2024-087374)
Supplement: Supplementary data [file bmjopen-2024-087374supp001.pdf]

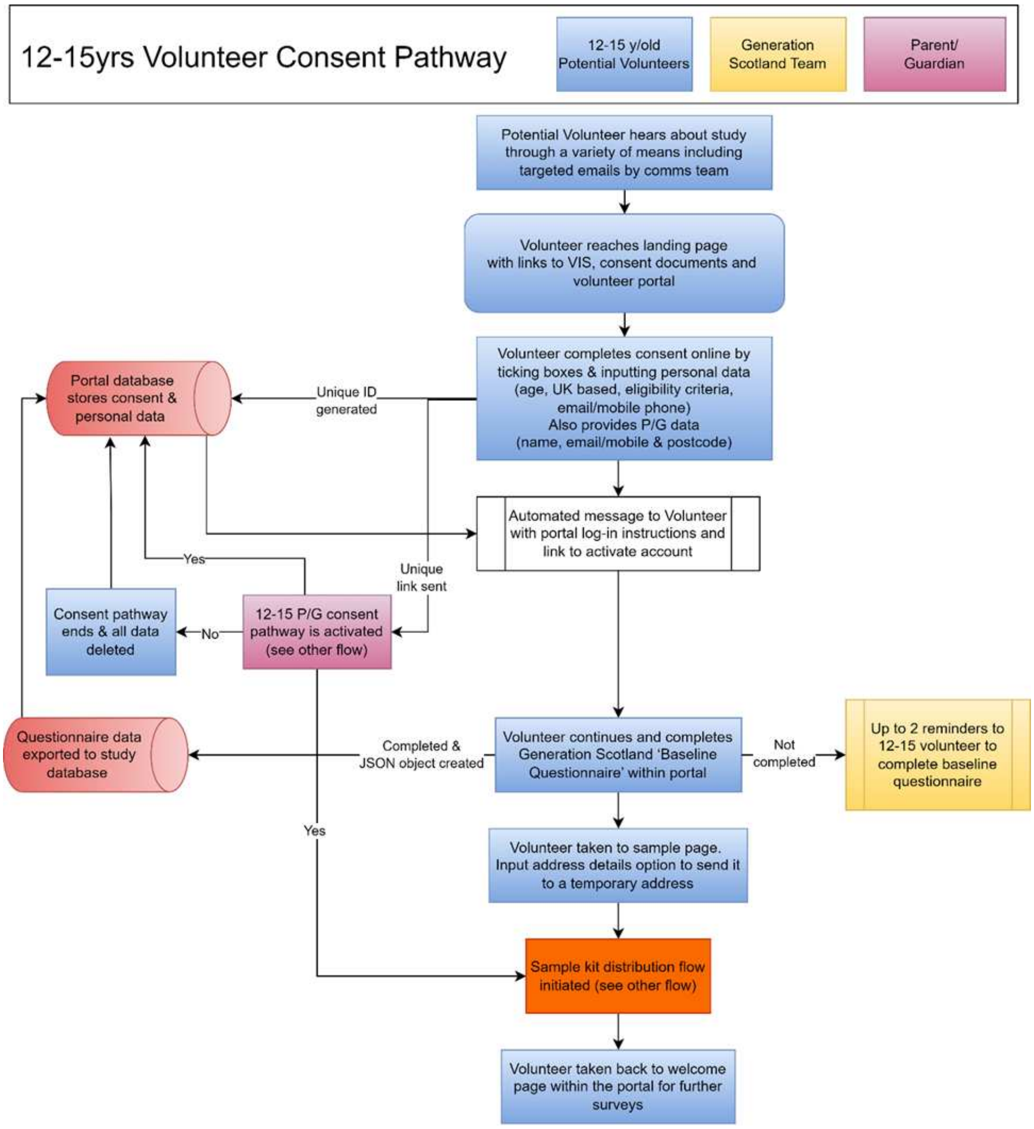

Figure.S1 12 – 15 years old volunteer consent pathway

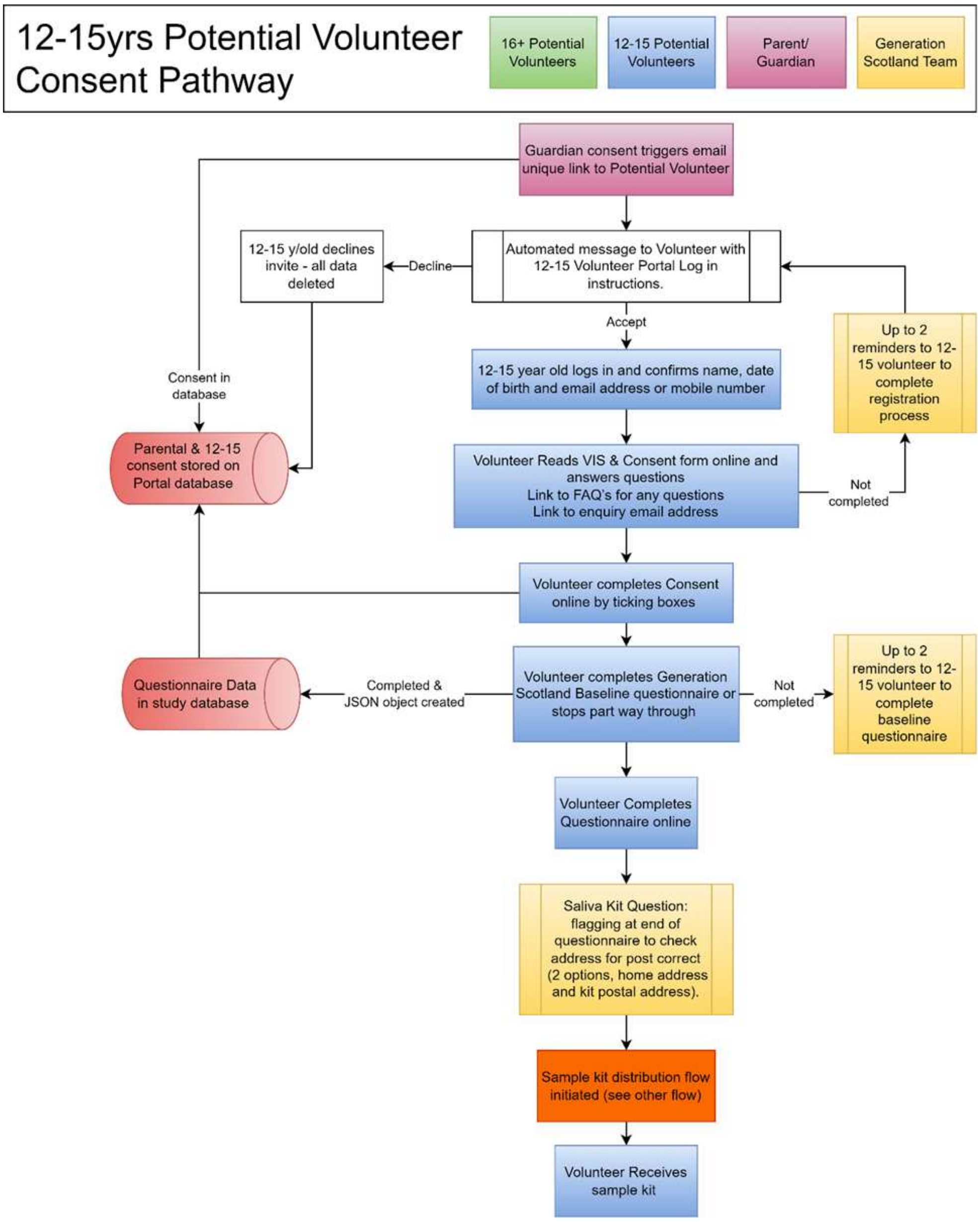

Figure.S2 12 – 15 years old potential volunteer consent pathway

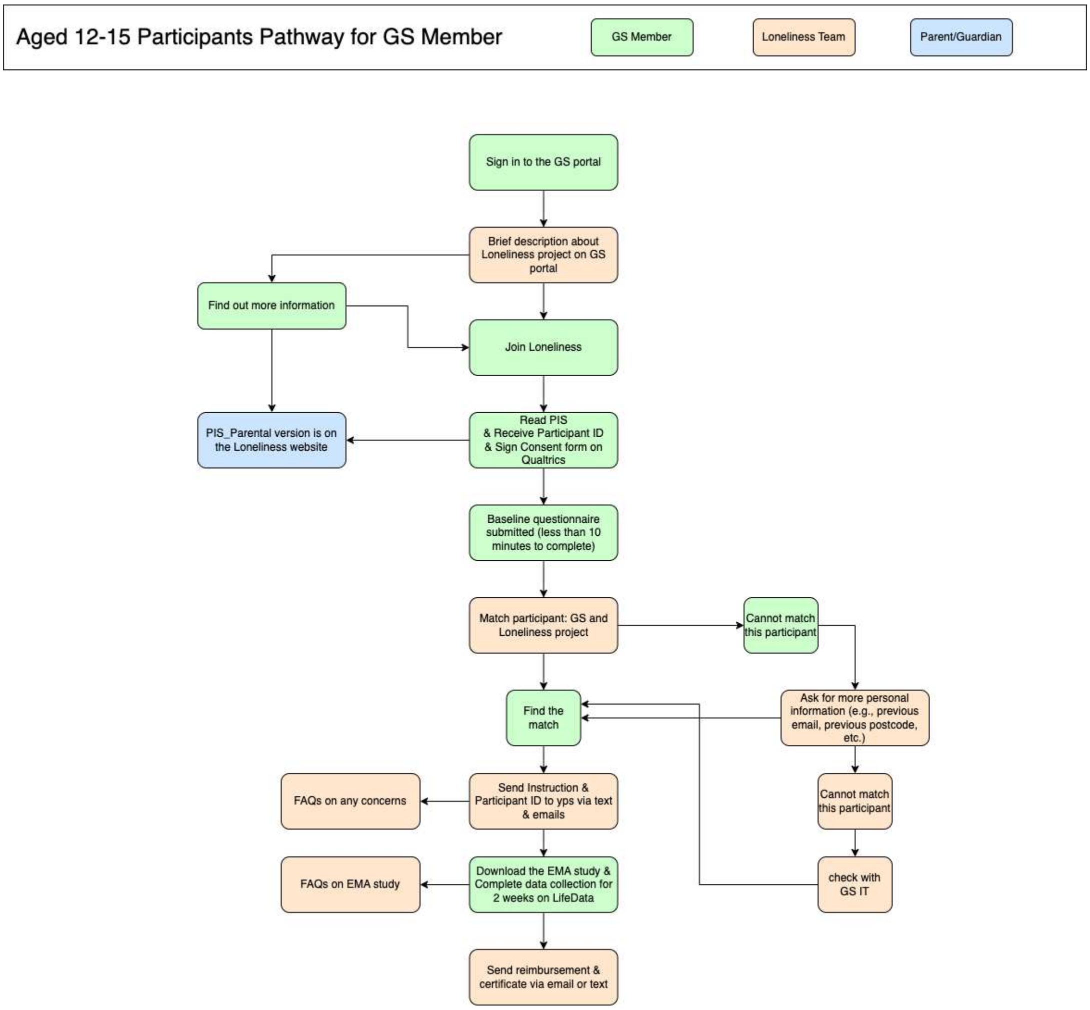

Figure.S3 Study Flow for young people who already are GS members

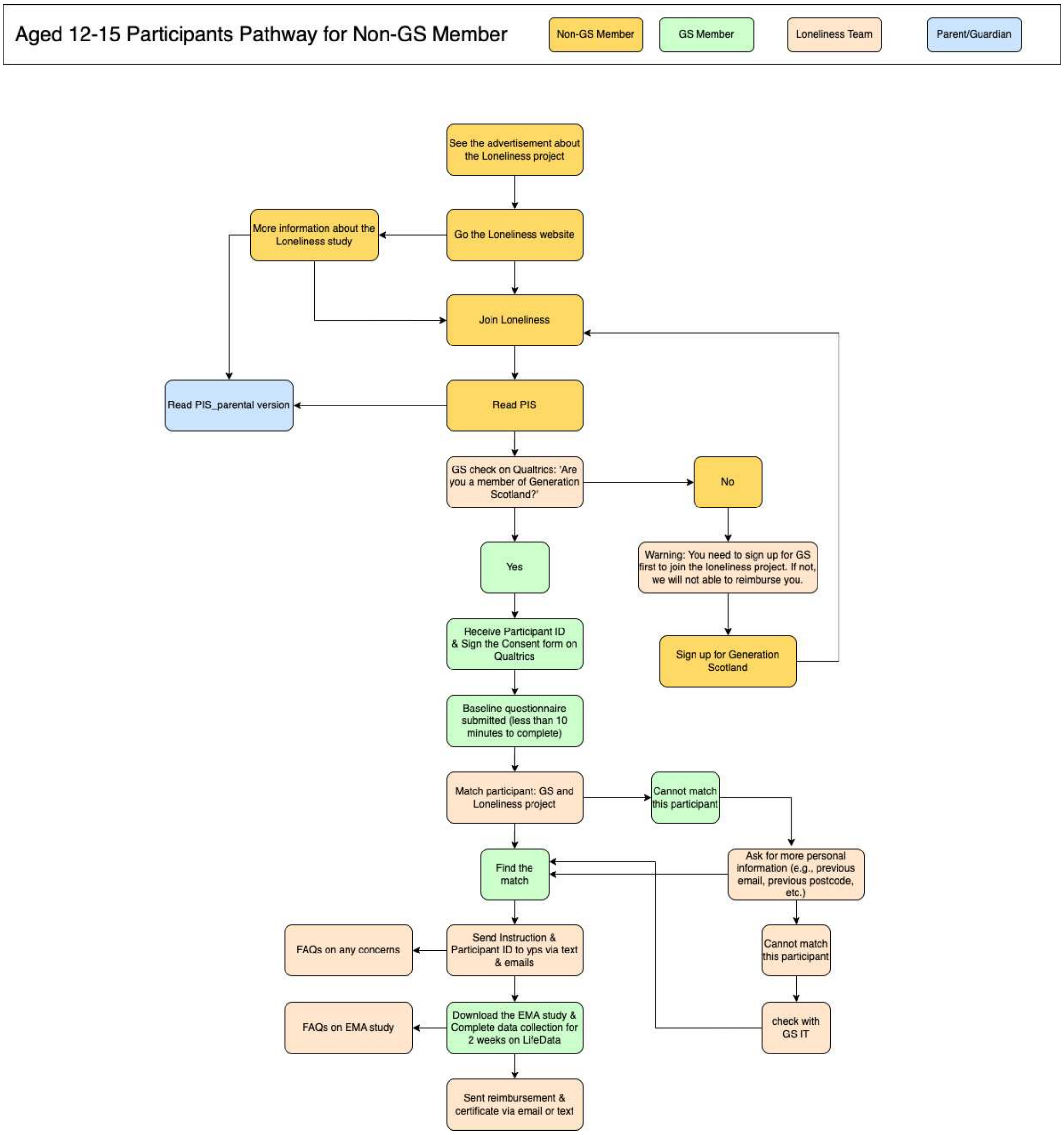

Figure.S4 Study Flow for young people who are not members of GS

Table S1. YPAG Involvement

	Characteristics	Methods	Content
Meeting 1<br>(4th April, 2023)	9 YP (girls: 5; boys: 4)<br>Mean age: 13.7y<br>Range: 11y – 17y	Online - Zoom	- What is a YPAG and why are we creating one (led by staff)<br>- Hopes for the YPAG (from YP)<br>- What Loneliness meant to you (from YP)<br>- Reimbursement approach (led by staff)<br>- Individuals task for next time (talk to your friends/family/teachers about what loneliness means to them)
Meeting 2<br>(9th May, 2023)	6 YP (girls: 4; boys: 2)<br>Mean age: 12.8y)<br>Range: 12y – 14y	Online – Zoom	- Review of previous meeting (led by staff)<br>- Presentations and discussion (from YP – what loneliness means to people)<br>- Introduce different types of loneliness and measures of loneliness (led by staff)<br>- Shared where you have your social interaction (from YP)<br>- Shared which types of social interaction you preferred (from YP)<br>- Tasks for next time (what loneliness means to people? Or where young people interact with others?)
Meeting 3<br>(30th May, 2023)	6 YP (girls: 4; boys: 2)<br>Mean age: 13.6y<br>Range: 12y – 15y	Online – Zoom	- Recap (led by staff)<br>- Overall aims of the loneliness project (led by staff)<br>- Group shared their presentation (from YP)<br>- Discussions around whether things have changed since and during lockdowns (led by staff and YP discussion)<br>- Brief overview of Generation Scotland (led by staff)<br>- Social Interaction and Loneliness Introduction (led by staff)
Meeting 4<br>(5th July, 2023)	6 YP (girls: 4; boys: 2)<br>Mean age: 13.6y<br>Range: 12y – 15y	Online – Zoom	- Introduction to ecological momentary assessment (led by staff)<br>- Discussion on EMA use (led by staff and YP discussion)<br>◊ Phones at school<br>◊ Length and frequency of prompts and questions<br>◊ Barriers<br>◊ Motivation<br>- Questionnaire and EMA feedback (from YP)

Meeting 5<br>(25th July, 2023)	3 YP (girls: 1; boys: 2)<br>Mean age: 13.3y<br>Range: 12y – 14y	Online – Zoom	- Recap (led by staff)<br>- Questionnaire results (led by staff and YP discussion)<br>◊ Number of prompts<br>◊ School hours<br>- Feedback on EMA design (from YP)
Meeting 6<br>(19th August, 2023)	7 YP (girls: 4; boys: 3)<br>Mean age: 14.2<br>Range: 11y – 17y	In-person – University Library	- Recap (led by staff)<br>- Piloting EMA (from YP)<br>- Feedback on EMA app (from YP)<br>- Feedback on baseline questionnaire (from YP)<br>- Loneliness artwork creation (from YP)<br>- Short video for social media platform – why joins this project (from YP)

Notes. YP = Young Person; YPAG = Young Person Advisory Group; EMA = Ecological Momentary Assessment.

Table S2. Measures for adolescent survey

Phase	Measures	Sources	Items, n
Baseline Questionnaire			
	Identifier	Postcode, phone number, email address	3
	Demographic	Age, sex, school transition, number of brothers and sisters, birth order, smoking in the past month, drinking in the past month, number of close friends	8
	Social Media Use	Smartphone access during weekday, smartphone access during weekend, average hours spent on social media during weekday, average hours spent on social media during weekend, how much time spent on direct message, passive message, passive in the public in the past hour, psychological aspect of social media use	10
	Attachment	Inventory of Parent and Peer Attachment (Buist et al., 2004)	11
	Depression & Anxiety	Revised Child Anxiety and Depression Scale (Chorpita et al., 2000)	16
	Personality	Ten-item Personality Inventory (Gosling, Rentfrow, & Swann Jr, 2003)	10
	Self-esteem	Rosenberg Self-Esteem Questionnaire (Rosenberg, 1965)	10

	Social Relationships	Social Relationships Questionnaire (Owens, & Hinshaw, 2016)	10
	Loneliness	UCLA (Russell, 1996)	20
	Perceived Social Support	Multidimensional Scale of Perceived Social Support (Zimet et al., 1988)	14
EMA Survey			
	In-person or Online Social Interaction	Have you had any online/in-person social interaction?	1
	Platform	Which social platform the interaction took place?	1
	Partner	Who was the main person this interaction was with?	1
	Feeling	Rejection/Acceptance, Pleasant/Unpleasant	2
	Positive Emotions	Adapted from Positive Affect Negative Affect Schedule expanded (Watson, & Clark, 1994)	3
	Negative Emotions	Adapted from Positive Affect Negative Affect Schedule expanded (Watson, & Clark, 1994)	3
	Loneliness	Adapted from UCLA (Russell, 1996)	3

Internalising Problems	Adapted from Revised Child Anxiety and Depression Scale (Chorpita et al., 2000)	6
Other Events	Any events that affected your emotions?	1

Notes. EMA = Ecological Momentary Assessment. The measurement for social media use was developed by the research team.
