# Supplementary material for: Loneliness in the Digital World: protocol for a co-produced ecological momentary assessment study in adolescents
Source: BMJ Open. 2024 Jun 6;14(6):e087374. doi: 10.1136/bmjopen-2024-087374 (PMC11163606; doi:10.1136/bmjopen-2024-087374)
Supplement: Supplementary data [file bmjopen-2024-087374supp002.pdf]

Total Prompt: 4 (daily) * 14 (days) = 56 prompts
Total items:
In-person Trigger: 22
Online Trigger: 24
No Interaction Trigger: 19

Start-up Block
Label: Informed Consent

You have already received information about this study and have agreed to take part. Please check the box below to confirm that this is the case.

If you have any questions, please email us at genscot_teens@ed.ad.uk, or text or call us at 07385 008242.

☐ I completed the consent form to take part in this study.

Label: Participant ID

Please enter your **Participant ID**

(The one you received from the previous survey)

Notification Initiated Session
[Block 1] Introduction – with graphic (56 prompt)

> Tell me about your most significant **Online** social interaction in the last hour. This can be any social interaction, big or small, positive or negative.
> Tell me about your most significant **In-person** social interaction in the last hour. This can be any social interaction, big or small, positive or negative.

[Block 2] Part 1: Interaction (56 prompts)

> Have you had any ONLINE social interaction in the last hour?
- Yes (Branch: 'Online')
- No, I didn't have online social interaction.
- No, but I had in-person social interaction. (Branch: 'In-person')

> Have you had any In-person social interaction in the last hour?
- Yes (Branch: 'In-person')
- No, I didn't have in-person social interaction
- No, but I had online social interaction. (Branch: 'Online')

Branch: 'Online':
Social platform (1 items – Multiple Choice):

> Please choose the App the interaction took place on…
- WhatsApp / SMS / Emails
- TikTok
- Snapchat
- YouTube

- Instagram
- Twitter (i.e., X)
- BeReal
- Discord
- Facebook
- Roblox
- Pinterest
- Amino
- Reddit
- Others (please specific)

Partner (1 items – Multiple Choice):

> Who was the main person this interaction was with?
- Friends
- Other Peers
- Family
- Known adults
- Someone not known to you
- Others (please specific)

Feeling (2 items – Multiple Choice)

> In relation to the person/people you had the interaction with…
To what extent did you feel:
- Strongly rejected
- Rejected
- Neither rejected nor accepted
- Accepted
- Strongly accepted
- Not applicable

> In relations to the person/people you had the interaction with…
To what extent did you feel:
- Strongly unpleasant
- Unpleasant
- Neither unpleasant nor pleasant
- Pleasant
- Strongly pleasant
- Not applicable

Branch: 'In-person':

Partner (1 items – Multiple Choice):

> Who was the main person this interaction was with?
- Friends
- Other Peers
- Family
- Known adults
- Someone not known to you
- Others (please specific)

Feeling (2 items – Multiple Choice)

> In relation to the person/people you had the interaction with…
To what extent did you feel:
- Strongly rejected
- Rejected
- Neither rejected nor accepted
- Accepted
- Strongly accepted
- Not applicable

> In relations to the person/people you had the interaction with…
To what extent did you feel:
- Strongly unpleasant
- Unpleasant
- Neither unpleasant nor pleasant
- Pleasant
- Strongly pleasant
- Not applicable

[Block 3] Introduction_Positive (~30 random character images)

> In this section, we will ask you questions related to **POSITIVE** emotions. Tell us, in the last hour, did you feel…

[Block 4] Positive Emotions (5 items – Multiple Choice)

> Happy
- Not at all
- Somewhat
- Moderately
- Very
- Extremely
- Not applicable

> Excited
- Not at all
- Somewhat
- Moderately
- Very
- Extremely
- Not applicable

> Enthusiastic
- Not at all
- Somewhat
- Moderately
- Very
- Extremely
- Not applicable

> Sense of belonging

- Not at all
- Somewhat
- Moderately
- Very
- Extremely
- Not applicable

> Determined

- Not at all
- Somewhat
- Moderately
- Very
- Extremely
- Not applicable

[Block 5] Introduction_Negative (~30 random character images)

> In this section, we will ask you questions related to **NEGATIVE** emotions. Tell us, in the last hour, did you feel…

[Block 6] Negative Emotions (5 items – Multiple Choice)

> Ashamed

- Not at all
- Somewhat
- Moderately
- Very
- Extremely
- Not applicable

> Nervous

- Not at all
- Somewhat
- Moderately
- Very
- Extremely
- Not applicable

> Distressed

- Not at all
- Somewhat
- Moderately
- Very
- Extremely
- Not applicable

> Upset

- Not at all
- Somewhat
- Moderately
- Very
- Extremely
- Not applicable

> Teased
- Not at all
- Somewhat
- Moderately
- Very
- Extremely
- Not applicable

[Block 7] Introduction_Loneliness (~30 random character images)

> In this section, we will ask you questions related to **LONELINESS**. Tell us, in the last hour, did you feel…

[Block 8] Loneliness (3 items – Multiple Choice)

> Lack of companionship
- Strongly agree
- Agree
- Neither agree nor disagree
- Strongly disagree
- Disagree
- Not applicable

> Left out
- Strongly agree
- Agree
- Neither agree nor disagree
- Strongly disagree
- Disagree
- Not applicable

> Isolated
- Strongly agree
- Agree
- Neither agree nor disagree
- Strongly disagree
- Disagree
- Not applicable

[Block 9] Anxiety (2 items – Multiple Choice)

> How anxious did you feel in the last hour?
- Not at all
- Somewhat
- Moderately
- Very
- Extremely
- Not applicable

> How worried did you feel in the last hour?
- Not at all
- Somewhat
- Moderately
- Very

- Extremely
- Not applicable

[Block 10] Depression (2 items – Multiple Choice)

> How low or depressed did you feel in the last hour?
- Not at all
- Somewhat
- Moderately
- Very
- Extremely
- Not applicable

> How hopeless did you feel in the last hour?
- Not at all
- Somewhat
- Moderately
- Very
- Extremely
- Not applicable

[Block 11] Unpleasant/Pleasant Events (1 item – Multiple Choice)

> In the last hour has anything happened that has affected your emotions positively or negatively that is not linked to social interaction (e.g., want to cinema, exam, missed the bus)
- Yes – Positive (Branch: 'Yes')
- Yes – Negative
- No

Branch Yes:

> How have these events affected your emotions?
- Not at all
- Somewhat
- Moderately
- Very
- Extremely
- Not applicable

[Block 12] Fun Stuff (~54 random images)

[Block 13] Reminder
