# Supplementary material for: Loneliness in the Digital World: protocol for a co-produced ecological momentary assessment study in adolescents
Source: BMJ Open. 2024 Jun 6;14(6):e087374. doi: 10.1136/bmjopen-2024-087374 (PMC11163606; doi:10.1136/bmjopen-2024-087374)
Supplement: Author's manuscript [file bmjopen-2024-087374.draft_revisions.pdf]

BMJ Open

BMJ Open is committed to open peer review. As part of this commitment we make the peer review history of every article we publish publicly available.

When an article is published we post the peer reviewers' comments and the authors' responses online. We also post the versions of the paper that were used during peer review. These are the versions that the peer review comments apply to.

The versions of the paper that follow are the versions that were submitted during the peer review process. They are not the versions of record or the final published versions. They should not be cited or distributed as the published version of this manuscript.

BMJ Open is an open access journal and the full, final, typeset and author-corrected version of record of the manuscript is available on our site with no access controls, subscription charges or pay-per-view fees (http://bmjopen.bmj.com).

If you have any questions on BMJ Open's open peer review process please email info.bmjopen@bmj.com

Loneliness in the Digital World: protocol for a co-produced Ecological Momentary Assessment study in adolescents

Journal:	*BMJ Open*
Manuscript ID	bmjopen-2024-087374
Article Type:	Protocol
Date Submitted by the Author:	08-Apr-2024

Complete List of Authors:	Xiao, Zhuoni; University of Edinburgh Western General Hospital, Centre for Genomic and Experimental Medicine<br>Robertson, Sarah; University of Edinburgh Western General Hospital<br>Long, Emily; University of Glasgow,<br>Flaig, Robin; University of Edinburgh Western General Hospital<br>Kirby, Liz; University of Edinburgh Western General Hospital<br>Romaniuk, Liana; University of Edinburgh, Division of Psychiatry<br>Murray, Aja; The University of Edinburgh, Department of Psychology<br>Whalley, Heather; University of Edinburgh, Centre for Clinical Brain Sciences Centre for Genomic and Experimental Medicine

Keywords:	Child & adolescent psychiatry < PSYCHIATRY, Psychometrics, MENTAL HEALTH

SCHOLARONE™
Manuscripts

Loneliness in the Digital World: protocol for a co-produced Ecological Momentary Assessment study in adolescents

Zhuoni Xiao[1,2]*, Sarah Robertson[1,2], Emily Long[3], Robin Flaig[1], Liz Kirby[1], Liana Romaniuk[2], Aja Murray[2,4Ω], Heather Whalley[1,2Ω]

[1] Generation Scotland, Centre for Genomic and Experimental Medicine, Institute of Genetics and Cancer, University of Edinburgh

[2] Division of Psychiatry, Centre for Clinical Brain Sciences, University of Edinburgh

[3] School of Health and Wellbeing, University of Glasgow

[4] Department of Psychology, University of Edinburgh

*Corresponding author at: Western General Hospital, Crewe Road, Edinburgh

email: zxiao@ed.ac.uk

Ω Authors contributed equally

For peer review only

Abstract

Introduction: Loneliness has been identified as an important public health issue, peaking during adolescence. Previous research has suggested that social interaction is a key factor in loneliness, and positive social interaction can act as a protective factor against loneliness. However, it is unclear whether there are differing impacts of in-person and online social interaction on adolescents' loneliness and mental health. Ecological Momentary Assessment (EMA) designs are ideally suited to better understanding these associations.

Method and Analysis: In the 'Loneliness in the Digital World' study, we will use a co-developed EMA design to capture daily social interactions, loneliness, and mental health such as positive and negative emotions, depression, and anxiety in approximately 200 adolescents aged 12 – 15 years. We will combine this with comprehensive information gathered from online surveys. Analysing the data using techniques such as dynamic structural equation modelling, we will examine, among other research questions, the associations between online and in-person social interaction and feelings of loneliness. The results can help inform interventions to support adolescents with high levels of loneliness and poor mental health.

Ethics and Dissemination: This study received a favourable ethical opinion through the ACCORD and College of Medicine & Veterinary Medicine Ethics panel from the University of Edinburgh. The results will be disseminated through journal publications, conferences, and seminar presentations and to relevant stakeholders such as teachers.

For peer review only

Strengthens and Limitations of this study

- ◊ This study will use an Ecological Momentary Assessment (EMA) design to collect near real-time data, capturing young people's social interaction experiences, loneliness, and moods in their natural environments. This approach minimizes recall bias and provides a more accurate reflection of daily life.
- ◊ This study used a co-production approach with a Young Person Advisory Group, who contributed to various aspects of the study design. This collaboration involved formulating research questions, determining measurements, , establishing an acceptable frequency of prompts for young people, creating appealing design of the app prompts, and deciding the optimal timing for prompt delivery.
- ◊ One potential limitation is that young people who agree to engage in EMA study may differ systematically from those who decline, including due to our instruction to consider not taking part if the topic is likely to trigger negative reactions.

For peer review only

Introduction

Loneliness has significant long-term consequences for physical and mental health (Park et al., 2020). Higher levels of loneliness predict physical health conditions, poorer mental health, more frequent sleep disturbances, as well as greater cognitive difficulties later in life (Lim, Eres, & Vasan, 2020; Heinrich & Gullone, 2006). Loneliness is especially salient for young people as they strive for independence and increasingly prioritize peer over familial relationships (Loades et al., 2020). Previous research has suggested that social interaction is a key factor in loneliness (Osborn, Weatherburn, & French, 2021). During adolescence young people become particularly sensitive to social rejection (Fuhrmann, Knoll, & Blakemore, 2015; Sebastian et al., 2011), placing them at a higher risk of loneliness (Pitman, Mann, & Johnson, 2018). The COVID-19 pandemic has further exacerbated this issue, with increased loneliness observed among younger ages since 2020 (Qualter et al., 2013; Van Tilburg et al., 2021).

Adolescence is a critical period that encompasses puberty, significant brain development (Fuhrmann et al., 2015), and an increased vulnerability for internalising problems such as social anxiety (see Rappe et al., 2019 for review). A previous meta-analysis conducted by Solmi et al. (2021) found that across 192 studies the peak age of onset of any mental disorders was age 14.5 years. However, many researchers focus on participants over 16 years, who are easier to recruit and consent. Hence, it is important to thoroughly investigate the understudied younger adolescent population. In this study, we will leverage our established connection with Generation Scotland to facilitate the recruitment of participants from this less-explored demographic. This strategic collaboration enables us to gain valuable insights into the loneliness and mental health of age group that has been previously overlooked relative to other age groups in this area of research.

For peer review only

An important consideration in studying loneliness is that young people are spending more time engaging with electronic devices and online interaction compared to previous generations where social interaction were more 'in-person'. Evidence on whether the amount of time young people spend online affects mental health is mixed. Indeed, there may be both risks and benefits (Kardefelt-Winther, Rees, & Livingstone, 2020). Some studies have suggested that the increase in social media use is linked to increase in loneliness and mental illness (Twenge, Spitzberg, & Campbell, 2019); however, others have suggested no meaningful associations (Jensen et al., 2019). This conflicting literature is likely due to limited understanding of how young people interact online, as well as limitations in the way that these interactions are measured. The impact of the digital world on young people's mental health remains unclear, necessitating nuanced research on online social interactions (Odger & Jensen, 2020).

Smartphone and social media usage are pervasive among adolescents, comprising the majority of their total screen time (Rideout et al., 2022; OfCom, U.K, 2022). In the UK, a substantial percentage (98%) of adolescents aged 12 - 15 have smartphones, and it is unsurprising that these devices were their most-used devices for accessing the internet (96%). Additionally, 93% of adolescents actively engage in social media, with WhatsApp being the most-used app among 12 – 15 (80%), followed by Snapchat (62%), Instagram (46%), and TikTok (44%) (OfCom UK, 2023). This is consistent with trends observed in other Western populations (Orben, 2020). Samples in the US and UK (2021-2022) estimate that the time adolescents spend on smartphones and social media ranges between one and a half hours to eight and a half hours per day (Kontostoli et al., 2023) with most adolescents spending between one and three hours per day (OfCom, U.K, 2023). Social media platforms provide nearly constant opportunities for individuals to connect and interact with others, irrespective of the time of day or geographical location (Naslund et al., 2020). Nonetheless, it remains unclear

For peer review only

whether online and offline social interaction have distinct effects on the levels of loneliness and mental health among adolescents.

The high levels of engagement of adolescents with digital technology can also be leveraged to gather high temporal resolution, ecologically valid research data to begin to address these questions. EMA has gained significant interest since the 2000s, with a notable increase in studies published annually (Wrzus & Neubauer, 2023). Encouragingly, qualitative data suggests that young people find EMA easy to engage with and report that it enhances their understanding of their own behaviours, feelings, and experiences (Acorda et al., 2021; Heron et al., 2017; Nap-van der Vlist et al., 2021; Russell & Gajos, 2020). Furthermore, a comprehensive review by Wen et al. (2017) examining compliance to EMA protocols in adolescents and children indicated that, across 42 studies, the weighted compliance rate stood at 78.3%. Moreover, there was no significant difference in compliance rates between studies involving clinical (76.9%) and non-clinical (79.2%) populations. Notably, among nonclinical studies, those prompting participants 2-3 times daily demonstrated a higher average compliance rate (91.7%) compared to studies with more frequent prompts (4-5 times: 77.4%; 6+: 75%). The reported compliance rates remained consistent across different durations of the EMA period in both clinical and nonclinical settings. Therefore, EMA holds promise as a method for studying young people's mental health, particularly in relation to their online world.

A crucial aspect of the study will involve developing best practice guidance on conducting Ecological Momentary Assessment in collaboration with the Young Person's Advisory Group (YPAG). YPAGs, conceptualised to enhance the acceptability and feasibility of research studies, play a crucial role in considering participants' perspectives and amplifying the relevance and impact of findings (Kirby, 2004). The increasing prioritisation of young people's voice, underscored by the UN Convention on the Rights of the Child, is now integrated into research guidance (Assembly, 1989). There is a growing emphasis on involving young

For peer review only

people as ‘co-actors’ in health research. Engaging in research brings various benefits for young people (Watson et al., 2023) as well, including meaningful impact, the opportunity to be part of a supportive community, chances for learning and growth, and increased opportunities overall. Consequently, involving YPAGs can be considered imperative for maximising the relevance and benefits of a research project in the realm of health research.

This study will explore how various types of social interaction, both positive and negative, impact state loneliness (i.e., a more temporary and situational experience of loneliness, which may be influenced by specific events or circumstances (Motta, 2021) and mental health in adolescents. We will use an EMA design to assess offline/online social acceptance and rejection in young people and examine how different types of social interaction affect state loneliness, emotions, and broader well-being.

Core research questions are:

- ◊ Does online social interaction and/or in-person social interaction predict later loneliness and mental health?
- ◊ Do online social interaction and in-person social interaction have the same associations to loneliness and mental health?
- ◊ Are there reciprocal relations among social interaction, loneliness, and mental health?
- ◊ Does the time of the day moderate these links?

The latter research question was suggested by the YPAG.

Exploratory analyses can be used to answer a range of additional questions such as exploration of moderation of the link between types of social interaction and loneliness by various other traits of young people. We will also develop best-practice standards for EMA research in young people, as well as validate the psychometric properties of EMA measurement tools.

For peer review only

Method and Analysis

Procedure

There are two phases in the current project – Phase 1: Young Person Advisory Group (YPAG) and Phase 2: full-scale study of 200 adolescents. Phase 1 is focused on conducting Patient and Public Involvement and Engagement (PPIE) work with the YPAG to co-produce a detailed protocol for the main EMA study. In Phase 2 we will launch the full EMA study, based on consultation with the YPAG.

Participants

Participants are expected to include approximately 200 adolescents aged 12 – 15 years from Scotland. Participants who take part in the current project will be sampled from the Generation Scotland (GS) study (Smith et al., 2013). GS is the largest family health study in Scotland and follows the health and well-being of multiple generations of families over time. Over 32,000 volunteers have already joined the study and helped research into cancer, diabetes, mental health, and other conditions (Smith et al., 2013). The current study is a new study embedded within GS. When young people join GS, their parents, or guardians have confirmed that they believe their young person has capacity to understand what is required to enrol in this study, and that this may involve follow-up studies approved by GS (See *Figure.S1* and *Figure.S2* for pathway for joining Generation Scotland in Supplementary Material A). The data gathered will be linked to participants' data from the main GS study. Participants will be offered a £20 e-voucher as compensation dependent on their response rates, as informed by the YPAG consultation processes. (See *Figure.S3* and *Figure.S4* for study flow in Supplementary Material A).

Inclusion and Exclusion criteria

For peer review only

Inclusion criteria for the young people will be: (1) aged 12 – 15 years old; (2) resident in Scotland; (3) Fluent in English; (4) Member of GS; (5) Able and willing to install LifeData (EMA data collection) App on their phone; if young people do not have access to a smartphone, our research team will provide one; (6) Ability to provide informed consent, and ability to independently complete online surveys and EMA protocols.

Since this study has the goal of encompassing a diverse population with various backgrounds, we have attempted to remove barriers to participation so that we can understand the broader impacts of social interaction on feelings of loneliness. Therefore, there will be no explicit exclusion criteria applied. However, given that some of the questions within the study (included aspects of social interaction, peer relations, and mood-related issues) have a small risk of triggering mental health crises, we highlight in the participant informant that young people who have experienced or are currently experiencing negative social interactions should carefully consider whether they wish to participate in the study.

Patient and Public Involvement and Engagement (PPIE)

Ten young people aged 12-15yrs from diverse backgrounds and locations across Scotland were recruited via different platforms. The opportunity was shared via Young Scot and YouthLink e-briefings, LGBT Youth Scotland Discord channel and existing youth work partners, including #iwill Scotland, RespectMe, Passion4Fusion, Glasgow Disability Alliance, Glasgow Young Movers, Glasgow Youth Work Network, Intergenerations Working Together, See Me Scotland and Salvesen Mindroom Centre. The School Health and Wellbeing Improvement Research Network (SHINE) promoted the opportunity to schools via their newsletter and we delivered a presentation at the SHINE annual conference. In addition, GS promoted the YPAG through posters which were shared on social media platforms including those most popular with young people, such as TikTok and Instagram. Six YPAG meetings

For peer review only

were held, and major changes were implemented based on their input to the protocol. For instance, YPAGs suggested that in-person social interactions are more salient than online social interaction; therefore, instead of asking the young people to recall their most impactful social interaction in the past hour, we now randomly ask them to recall their most impactful in-person or online social interaction in the past hour. Additionally, changes were made based on their input, such as reducing the length of the surveys, suggesting the time for each prompt, adding a progress bar for EMA surveys, incorporating a 'not applicable' option for all items, and embedding more fun and educational elements into the study. These elements include providing cartoon characters for each introduction, offering 'Fun Images' and 'Brain Facts' at the end of each completed EMA survey. As per the recommendations by Sellars et al. (2021) regarding consistent reporting of YPAG involvement, YPAG characteristics, frequency of engagement, methods and extent of involvement, parental engagement, and recognition of involvement are presented in Table S1 of the Supplementary Material A.

Data Collection

Data collection will begin in April 2024, and be completed in six months. *Figure.1* summarises the study flow. Informed consent will be collected from the young people online, and then they will be invited to complete an online baseline survey.

On completion of the online baseline survey, participants will initiate the EMA surveys. Once researchers confirmed they are members of GS, participants will be provided with a set of instructions for downloading the App, and offered on-boarding support via email, text, or call. Participants will use their own smartphone where possible; however, to prevent selection biases and encourage inclusivity, study-provided smartphones will be made accessible. An email helpline and phone number will also be available for participants to receive help on any issues they encounter.

For peer review only

EMA data collection will take place over a two-week period and involve four prompts per day at 8:00 – 9:00 am, 12:30 – 1:30 pm, 5:30 – 6:30 pm, and 10:00 – 11:00 pm. All of these will be administered via a smartphone application provided by LifeData. All measures will be designed to take less than one minute as lengthy questionnaires are linked to heightened burden and decreased compliance (Eisele et al., 2022). Consistent with this, our YPAG emphasised the importance of minimising burden.

At the end of the two-week EMA period, participants will be offered an e-voucher worth up to £20, with the exact amount to be dependent on their response rates (£5 for completion of baseline questionnaire and different amounts for EMA protocol depend on completion rate - £15 for 70% completion rate; £10 for 50% completion rate; £5 for 30% completion rate; £2 for under 30% completion rate). They will also receive a participation certificate. EMA surveys are provided in the online Supplementary Materials B. *Figure.2* presents the screenshots of the EMA protocol on phone.

Measures

The baseline questionnaire will be collected online via Qualtrics, and the EMA survey will be collected via the LifeData app. The measures that young people will complete over the course of the study are presented in Table 1.

Statistical Procedure

Data will be analysed using Dynamic Structural Equation Modelling (DSEM) (Asparouhov, Hamaker, & Muthén, 2018; Asparouhov, & Muthén, 2020; McNeish, & Hamaker, 2020). DSEM combines multi-level structural equation, and time-series modelling, which allows an examination of individual differences in within-person dynamics such as the connections between social interaction and emotions. We will utilize a multilevel CFA model to examine the psychometric properties of a three-item loneliness measurement. Then, we will

For peer review only

use DSEM with lag-1 autoregressive and cross-lagged effects to examine the impacts of social rejection/acceptance on itself over time, and loneliness or emotions at a later time. We will also utilize mediation within DSEM to explore whether state emotions mediate the associations between social interaction and loneliness or mental wellbeing. We will using time-varying effects DSEM to investigate whether time of the day moderate these associations. These models will be implemented in the latest available versions of Mplus and R.

Sample Size Justification

We will use a sample size of N = 200, adolescents aged 12 to 15 years old. Power analyses are based on a Monte Carlo approach, setting a threshold of $\alpha = 0.05$ and $1- \beta = .80$. Using this approach, the sample size necessary to detect significant effects in key parameters (e.g., moderation of a cross-lagged within-person effect) based on realistic parameter estimates was explored. These realistic parameter estimates were taken from DSEM models fit to past similar EMA data by Murray and team (Murray et al., 2022; Murray et al., 2023). We determined that an N = 100 would provide sufficient power. Therefore, a sample size of N = 200 would provide a strong buffer against attrition or missingness, as well as allow us to examine sub-groups such as by gender.

Ethics and Dissemination.

Ethics review for the data collection will first obtain sponsorship review from ACCORD, and then will be by the Collee of Medicine & Veterinary Medicine Ethics panel. The results will be disseminated through journal publication, conferences, and seminar presentations.

Discussion

The current protocol describes the design for a joint traditional and EMA survey study of the daily life social interactions and their impacts of adolescents' loneliness and mental

wellbeing. We will build capacity and guidance on conducting robust, youth-led research on adolescent mental health, through engagement and methodological innovation. Collaborating with young people aged 12 to 15 years, we will help contribute to best-practice standards for EMA research, psychometrically validated measurement tools, and apply cutting-edge analysis methods for these complex data. We will gather information on whether different types of social interactions have the same impacts on loneliness and mental wellbeing, whether there are gender differences in these associations, whether social rejection affects subsequent emotions and loneliness, and whether current social acceptance would help recovery from previous social rejection by reducing loneliness or improving mental wellbeing. Our findings can therefore inform better understanding of the impacts of social interaction on loneliness and illuminate poorly understood areas of adolescent mental health with implications for policy and clinical practice. For example, the World Health Organisation Commission on Social Connection (2024 – 2026) aims to see loneliness be recognised and resourced as a global public health priority. Our research could provide preliminary findings for developing solutions for loneliness, as well as valid measurements for assessing loneliness as it plays out in real-time. Our findings can help inform, for example, prescribe culture (e.g., better peer relations or better online social behaviours to improve mental health and wellbeing).

Previous interventions for loneliness focus on different aspects. For instance, social skills (Barry et al., 2003; Beidel, Turner, & Morris, 2000; Bostick & Anderson, 2009; King et al., 1997), social interaction (Battles & Wiener, 2002; Grace et al., 2014; Lim et al., 2019), emotional skills (Bostick & Anderson, 2009), enhanced social support (Brouzon, Vassilopoulos, & Moschou, 2016; Bradley, 2016; Kneer et al., 2019; Steward et al., 2011; Stewart et al., 2013) or psychological interventions (Brouzon, Vassilopoulos, & Moschou, 2016; Smith et al., 2017). However, most interventions are targeted at adults, older adults, or patients' groups, rather than tailored for the needs adolescents. Findings from the current

For peer review only

project could inform interventions tailored for adolescents and that reflect their social interaction habits such as promoting healthy online behaviours.

Our methodology has the potential to accelerate advancements in the utilization of Ecological Momentary Assessment (EMA) technique within adolescent populations in school setting. Through our research, we aim to investigate the impact of prompt timing on response rates, specifically exploring whether prompts during school hours versus after-school hours influence response rates. By providing a template for such research (which we will make openly available in the form of openly accessible study materials, including measures and analysis code), and systematically recording challenges, solutions, and advantages, this study can foster broader adoption of EMA methodologies.

Limitations

While extensive research has explored optimal design features for Ecological Momentary Assessment (EMA) in general (Eisele et al., 2022; Rintala et al., 2020), the relative novelty of the approach for measuring social interactions in adolescents means there is limited past research to guide key methodological decisions such as measure selection, development or validation, sampling frequency and schedules, reimbursement, and strategies to promote engagement (Murray, & Xie, 2023). There may also be difficulties in gathering information during school hours meaning that we may have high levels of missingness during the day. Finally, the sample will be drawn from the GS study. While this has the benefit of providing comprehensive measures to link to the EMA data to, the sample may be selective in the sense that they have demonstrated a motivation to engage in health research.

For peer review only

Reference:

Acorda, D., Businelle, M., & Santa Maria, D. (2021). Perceived impacts, acceptability, and recommendations for ecological momentary assessment among youth experiencing homelessness: qualitative study. *JMIR Formative Research*, *5*(4), e21638.

Asparouhov, T., & Muthén, B. (2020). Comparison of models for the analysis of intensive longitudinal data. *Structural Equation Modeling: A Multidisciplinary Journal*, *27*(2), 275-297.

Asparouhov, T., Hamaker, E. L., & Muthén, B. (2018). Dynamic structural equation models. *Structural equation modeling: a multidisciplinary journal*, *25*(3), 359-388.

Assembly, U. G. (1989). Convention on the Rights of the Child. *United Nations, Treaty Series*, *1577*(3), 1-23.

Barry, T. D., Klinger, L. G., Lee, J. M., Palardy, N., Gilmore, T., & Bodin, S. D. (2003). Examining the effectiveness of an outpatient clinic–based social skills group for high-functioning children with autism. *Journal of autism and developmental disorders*, *33*, 685-701.

Battles, H. B., & Wiener, L. S. (2002). STARBRIGHT World: Effects of an electronic network on the social environment of children with life-threatening illnesses. *Children's Health Care*, *31*(1), 47-68.

Beidel, D. C., Turner, S. M., & Morris, T. L. (2000). Behavioral treatment of childhood social phobia. *Journal of consulting and clinical psychology*, *68*(6), 1072.

Bostick, D., & Anderson, R. (2009). Evaluating a small-group counseling program—A model for program planning and improvement in the elementary setting. *Professional School Counseling*, *12*(6), 2156759X0901200602.

For peer review only

Bradley, R. (2016). ‘Why single me out?’Peer mentoring, autism and inclusion in mainstream secondary schools. *British Journal of Special Education*, *43*(3), 272-288.

Brouzos, A., Vassilopoulos, S. P., & Moschou, K. (2016). Utilizing storytelling to promote emotional well-being of children with a distinct physical appearance: The case of children who wear eyeglasses. *The European Journal of Counselling Psychology*, *4*(1).

Buist, K. L., Deković, M., Meeus, W. H., & van Aken, M. A. G. (2004). Attachment in Adolescence:: A Social Relations Model Analysis. Journal of Adolescent Research, 19(6), 826-850.

Chorpita, B. F., Yim, L., Moffitt, C., Umemoto, L. A., & Francis, S. E. (2000). Assessment of symptoms of DSM-IV anxiety and depression in children: A revised child anxiety and depression scale. Behaviour research and therapy, 38(8), 835-855.

Eisele, G., Vachon, H., Lafit, G., Kuppens, P., Houben, M., Myin-Germeys, I., & Viechtbauer, W. (2022). The effects of sampling frequency and questionnaire length on perceived burden, compliance, and careless responding in experience sampling data in a student population. *Assessment*, *29*(2), 136-151.

Eisele, G., Vachon, H., Lafit, G., Kuppens, P., Houben, M., Myin-Germeys, I., & Viechtbauer, W. (2022). The effects of sampling frequency and questionnaire length on perceived burden, compliance, and careless responding in experience sampling data in a student population. *Assessment*, *29*(2), 136-151.

Fuhrmann, D., Knoll, L. J., & Blakemore, S. J. (2015). Adolescence as a sensitive period of brain development. *Trends in cognitive sciences*, *19*(10), 558-566.

Gosling, S. D., Rentfrow, P. J., & Swann Jr, W. B. (2003). A very brief measure of the Big-Five personality domains. Journal of Research in personality, 37(6), 504-528.

For peer review only

Grace, E., Raghavendra, P., Newman, L., Wood, D., & Connell, T. (2014). Learning to use the Internet and online social media: What is the effectiveness of home-based intervention for youth with complex communication needs?. *Child Language Teaching and Therapy*, *30*(2), 141-157.

Hankin, B. L., Young, J. F., Abela, J. R., Smolen, A., Jenness, J. L., Gulley, L. D., ... & Oppenheimer, C. W. (2015). Depression from childhood into late adolescence: Influence of gender, development, genetic susceptibility, and peer stress. *Journal of abnormal psychology*, *124*(4), 803.

Heinrich, L. M., & Gullone, E. (2006). The clinical significance of loneliness: A literature review. *Clinical psychology review*, *26*(6), 695-718.

Heinrich, L. M., & Gullone, E. (2006). The clinical significance of loneliness: A literature review. *Clinical psychology review*, *26*(6), 695-718.

Heron, K. E., & Smyth, J. M. (2010). Ecological momentary interventions: incorporating mobile technology into psychosocial and health behaviour treatments. *British journal of health psychology*, *15*(1), 1-39.

Heron, K. E., Everhart, R. S., McHale, S. M., & Smyth, J. M. (2017). Using mobile-technology-based ecological momentary assessment (EMA) methods with youth: A systematic review and recommendations. *Journal of pediatric psychology*, *42*(10), 1087-1107.

Jensen, M., George, M. J., Russell, M. R., & Odgers, C. L. (2019). Young adolescents' digital technology use and mental health symptoms: Little evidence of longitudinal or daily linkages. *Clinical Psychological Science*, *7*(6), 1416-1433.

Kardefelt-Winther, D., Rees, G., & Livingstone, S. (2020). Contextualising the link between adolescents' use of digital technology and their mental health: A multi-country study

of time spent online and life satisfaction. *Journal of Child Psychology and Psychiatry*, *61*(8), 875-889.

King, G. A., Specht, J. A., Schultz, I., Warr-Leeper, G., Redekop, W., & Risebrough, N. (1997). Social skills training for withdrawn unpopular children with physical disabilities: A preliminary evaluation. *Rehabilitation Psychology*, *42*(1), 47.

Kirby, P. (2004). A guide to actively involving young people in research. *For Researchers, Research Commissioners and Managers, Involve Support Unit, Eastleigh, Hamphires*.

Kneer, J., van Eldik, A., Jansz, J., Eischeid, S., & Usta, M. (2019). With a little help from my friends: Peer coaching for refugee adolescents and the role of social media. *Media and Communication*, *7*(2), 264-274.

Kontostoli, E., Jones, A. P., Pearson, N., Foley, L., Biddle, S. J., & Atkin, A. J. (2023). The association of contemporary screen behaviours with physical activity, sedentary behaviour and sleep in adolescents: A cross-sectional analysis of the millennium cohort study. *International Journal of Behavioral Medicine*, *30*(1), 122-132.

Kwong, A. S., Manley, D., Timpson, N. J., Pearson, R. M., Heron, J., Sallis, H., ... & Leckie, G. (2019). Identifying critical points of trajectories of depressive symptoms from childhood to young adulthood. *Journal of youth and adolescence*, *48*, 815-827.

Lim, M. H., Eres, R., & Vasan, S. (2020). Understanding loneliness in the twenty-first century: an update on correlates, risk factors, and potential solutions. *Social psychiatry and psychiatric epidemiology*, *55*, 793-810.

Lim, M. H., Eres, R., & Vasan, S. (2020). Understanding loneliness in the twenty-first century: an update on correlates, risk factors, and potential solutions. *Social psychiatry and psychiatric epidemiology*, *55*, 793-810.

For peer review only

Lim, M. H., Rodebaugh, T. L., Eres, R., Long, K. M., Penn, D. L., & Gleeson, J. F. (2019). A pilot digital intervention targeting loneliness in youth mental health. *Frontiers in psychiatry*, *10*, 604.

Loades, M. E., Chatburn, E., Higson-Sweeney, N., Reynolds, S., Shafran, R., Brigden, A., ... & Crawley, E. (2020). Rapid systematic review: the impact of social isolation and loneliness on the mental health of children and adolescents in the context of COVID-19. *Journal of the American Academy of Child & Adolescent Psychiatry*, *59*(11), 1218-1239.

McNeish, D., & Hamaker, E. L. (2020). A primer on two-level dynamic structural equation models for intensive longitudinal data in Mplus. *Psychological methods*, *25*(5), 610.

Murray, A. L., & Xie, T. (2023). Engaging adolescents in contemporary longitudinal health research: Strategies for promoting participation and retention. *Journal of Adolescent Health*.

Murray, A. L., Speyer, L. G., Brown, R., Zhu, X., Yang, Y., Eisner, M., & Ribeaud, D. (2022). Advancing multi-timeframe developmental research through combining long-term cohort and ecological momentary assessment studies: The Decades-to-minutes (D2M) study.

Murray, A., Ushakova, A., Zhu, X., Yang, Y., Xiao, Z., Brown, R., ... & Eisner, M. (2023). Predicting Participation Willingness in Ecological Momentary Assessment of General Population Health and Behavior: Machine Learning Study. *Journal of medical Internet research*, *25*, e41412.

Nap-van der Vlist, M. M., Houtveen, J., Dalmeijer, G. W., Grootenhuis, M. A., van der Ent, C. K., van Grotel, M., ... & Nijhof, S. L. (2021). Internet and smartphone-based ecological

For peer review only

momentary assessment and personalized advice (PROfeel) in adolescents with chronic conditions: a feasibility study. *Internet Interventions*, *25*, 100395.

Naslund, J. A., Bondre, A., Torous, J., & Aschbrenner, K. A. (2020). Social media and mental health: benefits, risks, and opportunities for research and practice. *Journal of technology in behavioral science*, *5*, 245-257.

Odgers, C. L., & Jensen, M. R. (2020). Annual research review: Adolescent mental health in the digital age: Facts, fears, and future directions. *Journal of Child Psychology and Psychiatry*, *61*(3), 336-348.

OfCom, U. K. (2022). Children and parents: Media use and attitudes report. *London: Office of Communications London*. Available: https://www.ofcom.org.uk/__data/assets/pdf_file/0024/234609/childrens-media-use-and-attitudes-report-2022.pdf

OfCom, U. K. (2023). Children and parents: Media use and attitudes report. *London: Office of Communications London*. Available: https://www.ofcom.org.uk/__data/assets/pdf_file/0027/255852/childrens-media-use-and-attitudes-report-2023.pdf

Orben, A. (2020). Teenagers, screens and social media: a narrative review of reviews and key studies. *Social psychiatry and psychiatric epidemiology*, *55*(4), 407-414.

Osborn, T., Weatherburn, P., & French, R. S. (2021). Interventions to address loneliness and social isolation in young people: A systematic review of the evidence on acceptability and effectiveness. *Journal of adolescence*, *93*, 53-79.

For peer review only

Owens, E. B., & Hinshaw, S. P. (2016). Childhood conduct problems and young adult outcomes among women with childhood attention-deficit/hyperactivity disorder (ADHD). *Journal of abnormal psychology*, *125*(2), 220.

Park, C., Majeed, A., Gill, H., Tamura, J., Ho, R. C., Mansur, R. B., ... & McIntyre, R. S. (2020). The effect of loneliness on distinct health outcomes: a comprehensive review and meta-analysis. *Psychiatry Research*, *294*, 113514.

Pitman, A., Mann, F., & Johnson, S. (2018). Advancing our understanding of loneliness and mental health problems in young people. *The Lancet Psychiatry*, *5*(12), 955-956.

Qualter, P., Brown, S. L., Rotenberg, K. J., Vanhalst, J., Harris, R. A., Goossens, L., ... & Munn, P. (2013). Trajectories of loneliness during childhood and adolescence: Predictors and health outcomes. *Journal of adolescence*, *36*(6), 1283-1293.

Rapee, R. M., Oar, E. L., Johnco, C. J., Forbes, M. K., Fardouly, J., Magson, N. R., & Richardson, C. E. (2019). Adolescent development and risk for the onset of social-emotional disorders: A review and conceptual model. *Behaviour research and therapy*, *123*, 103501.

Rideout, V., Peebles, A., Mann, S., & Robb, M. B. (2022). Common Sense census: Media use by tweens and teens, 2021. Common Sense.

Rintala, A., Wampers, M., Myin-Germeys, I., & Viechtbauer, W. (2020). Momentary predictors of compliance in studies using the experience sampling method. *Psychiatry research*, *286*, 112896.

Rosenberg, M. (1965). Rosenberg self-esteem scale (RSE). Acceptance and commitment therapy. Measures package, 61(52), 18.

Russell, D. W. (1996). UCLA Loneliness Scale (Version 3): Reliability, validity, and factor structure. Journal of personality assessment, 66(1), 20-40.

Russell, M. A., & Gajos, J. M. (2020). Annual Research Review: Ecological momentary assessment studies in child psychology and psychiatry. *Journal of Child Psychology and Psychiatry*, *61*(3), 376-394.

Sebastian, C. L., Tan, G. C., Roiser, J. P., Viding, E., Dumontheil, I., & Blakemore, S. J. (2011). Developmental influences on the neural bases of responses to social rejection: implications of social neuroscience for education. *Neuroimage*, *57*(3), 686-694.

Sellars, E., Pavarini, G., Michelson, D., Creswell, C., & Fazel, M. (2021). Young people's advisory groups in health research: scoping review and mapping of practices. *Archives of disease in childhood*, *106*(7), 698-704.

Smith, B. H., Campbell, A., Linksted, P., Fitzpatrick, B., Jackson, C., Kerr, S. M., ... & Morris, A. D. (2013). Cohort Profile: Generation Scotland: Scottish Family Health Study (GS: SFHS). The study, its participants and their potential for genetic research on health and illness. *International journal of epidemiology*, *42*(3), 689-700.

Smith, N. G., Hart, T. A., Kidwai, A., Vernon, J. R., Blais, M., & Adam, B. (2017). Results of a pilot study to ameliorate psychological and behavioral outcomes of minority stress among young gay and bisexual men. *Behavior therapy*, *48*(5), 664-677.

Solmi, M., Radua, J., Olivola, M., Croce, E., Soardo, L., Salazar de Pablo, G., ... & Fusar-Poli, P. (2022). Age at onset of mental disorders worldwide: large-scale meta-analysis of 192 epidemiological studies. *Molecular psychiatry*, *27*(1), 281-295.

For peer review only

Stewart, M., Barnfather, A., Magill-Evans, J., Ray, L., & Letourneau, N. (2011). Brief report: An online support intervention: Perceptions of adolescents with physical disabilities. *Journal of adolescence*, *34*(4), 795-800.

Twenge, J. M., Spitzberg, B. H., & Campbell, W. K. (2019). Less in-person social interaction with peers among US adolescents in the 21st century and links to loneliness. *Journal of Social and Personal Relationships*, *36*(6), 1892-1913.

Van Tilburg, T. G., Steinmetz, S., Stolte, E., Van der Roest, H., & de Vries, D. H. (2021). Loneliness and mental health during the COVID-19 pandemic: A study among Dutch older adults. *The Journals of Gerontology: Series B*, *76*(7), e249-e255.

Watson, D., & Clark, L. A. (1994). The PANAS-X: Manual for the positive and negative affect schedule-expanded form.

Watson, R., Burgess, L., Sellars, E., Crooks, J., McGowan, R., Diffey, J., ... & McMellon, C. (2023). A qualitative study exploring the benefits of involving young people in mental health research. *Health Expectations*.

Wen, C. K. F., Schneider, S., Stone, A. A., & Spruijt-Metz, D. (2017). Compliance with mobile ecological momentary assessment protocols in children and adolescents: a systematic review and meta-analysis. *Journal of medical Internet research*, *19*(4), e132.

Wrzus, C., & Neubauer, A. B. (2023). Ecological momentary assessment: A meta-analysis on designs, samples, and compliance across research fields. *Assessment*, *30*(3), 825-846.

Zimet, G. D., Dahlem, N. W., Zimet, S. G., & Farley, G. K. (1988). The multidimensional scale of perceived social support. Journal of personality assessment, 52(1), 30-41.

For peer review only

Authors' contributions

Conceptualisation: HW. Funding acquisition: HW. Methodology: ZX, AM and HW. Writing – original draft: ZX. Writing – review and editing: ZX, SR,EL, RF, LR, AM and HW.

Funding Statement

The research describe within this report was supported by Medical Research Council (MRC) grant (Grant Ref: MR/X002608/1), and AM was additionally supported by a Wellcome Trust Career Development Grant (REF: 225364/Z/22/Z).

Competing Interests Statement

N/A

For peer review only

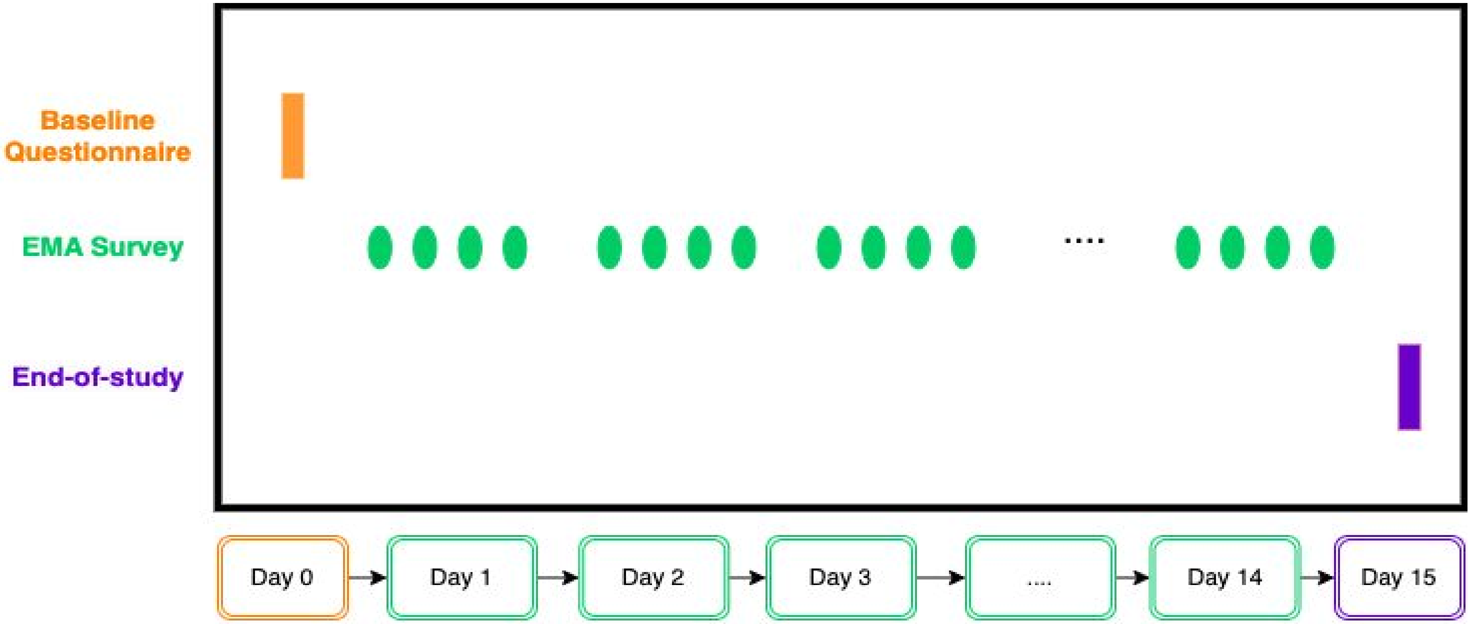

Figure.1 Summary of data collection schedule. EMA = Ecological Momentary Assessment.

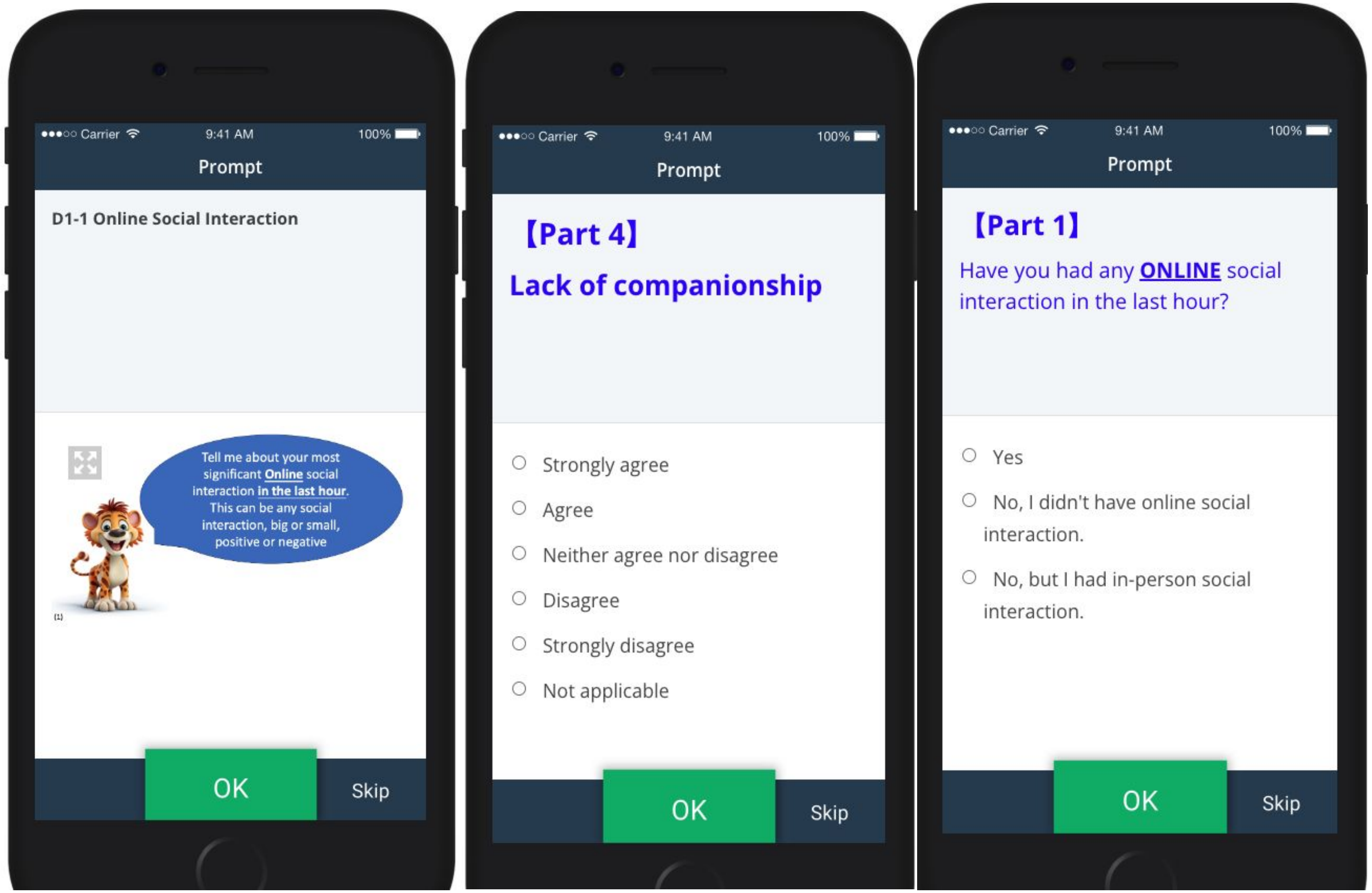

Figure.2 Screenshots of EMA protocol on phone

Table 1. Measures for adolescent survey

Phase	Measures	Sources	Items, n
Baseline Questionnaire			
	Identifier	Postcode, phone number, email address	3
	Demographic	Age, sex, school transition, number of brothers and sisters, birth order, smoking in the past month, drinking in the past month, number of close friends	8
	Social Media Use	Smartphone access during weekday, smartphone access during weekend, average hours spent on social media during weekday, average hours spent on social media during weekend, how much time spent on direct message, passive message, passive in the public in the past hour, psychological aspect of social media use	10
	Attachment	Inventory of Parent and Peer Attachment (Buist et al., 2004)	11
	Depression & Anxiety	Revised Child Anxiety and Depression Scale (Chorpita et al., 2000)	16
	Personality	Ten-item Personality Inventory (Gosling, Rentfrow, & Swann Jr, 2003)	10

For peer review only

	Self-esteem	Rosenberg Self-Esteem Questionnaire (Rosenberg, 1965)	10
	Social Relationships	Social Relationships Questionnaire (Owens, & Hinshaw, 2016)	10
	Loneliness	UCLA (Russell, 1996)	20
	Perceived Social Support	Multidimensional Scale of Perceived Social Support (Zimet et al., 1988)	14
EMA Survey			
	In-person or Online Social Interaction	Have you had any online/in-person social interaction?	1
	Platform	Which social platform the interaction took place?	1
	Partner	Who was the main person this interaction was with?	1
	Feeling	Rejection/Acceptance, Pleasant/Unpleasant	2
	Positive Emotions	Adapted from Positive Affect Negative Affect Schedule expanded (Watson, & Clark, 1994)	3
	Negative Emotions	Adapted from Positive Affect Negative Affect Schedule expanded (Watson, & Clark, 1994)	3
	Loneliness	Adapted from UCLA (Russell, 1996)	3

For peer review only

Internalising Problems	Adapted from Revised Child Anxiety and Depression Scale (Chorpita et al., 2000)	6
Other Events	Any events that affected your emotions?	1

Notes. EMA = Ecological Momentary Assessment. The measurement for social media use was developed by the research team.

For peer review only

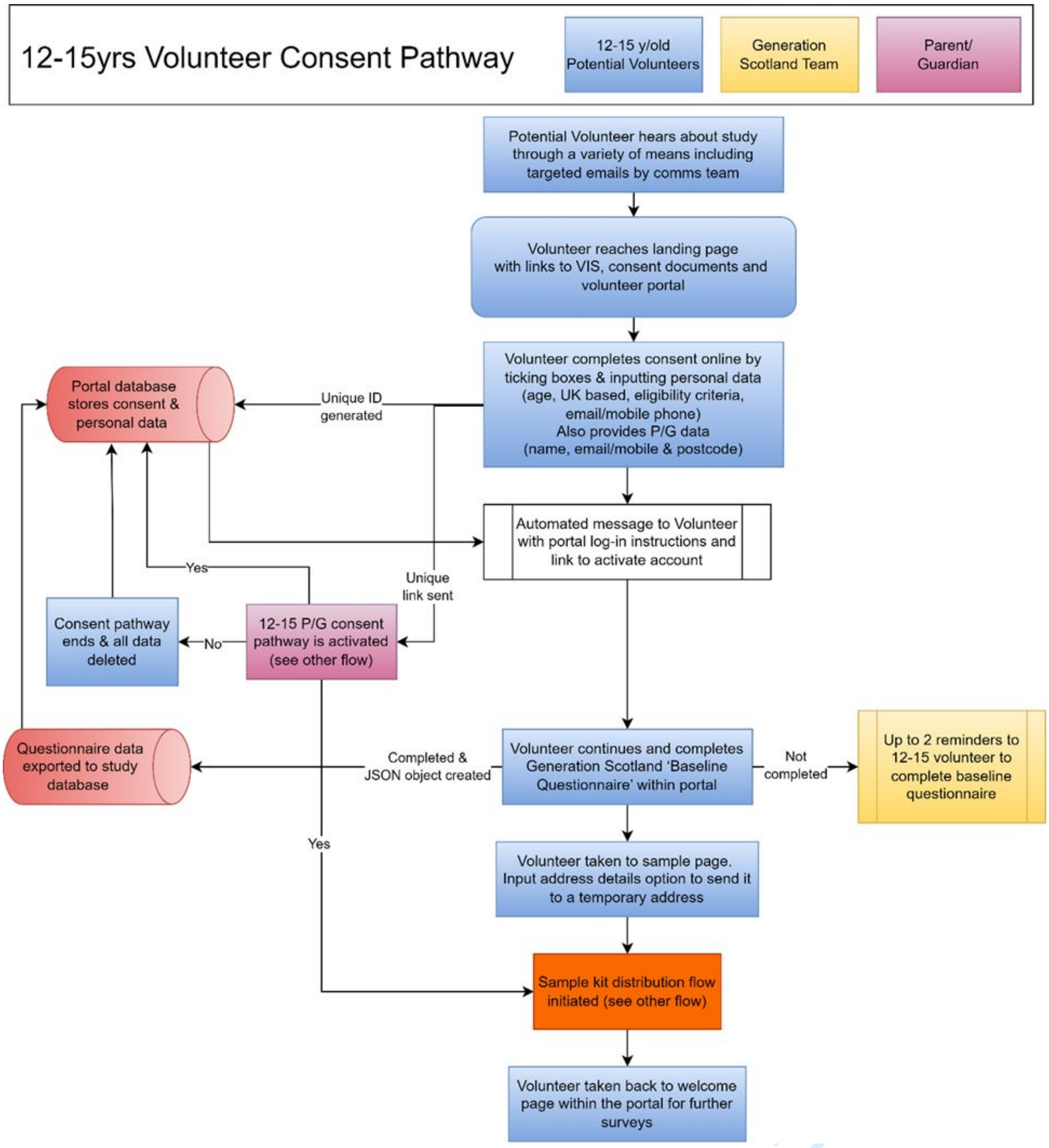

Figure.S1 12 – 15 years old volunteer consent pathway

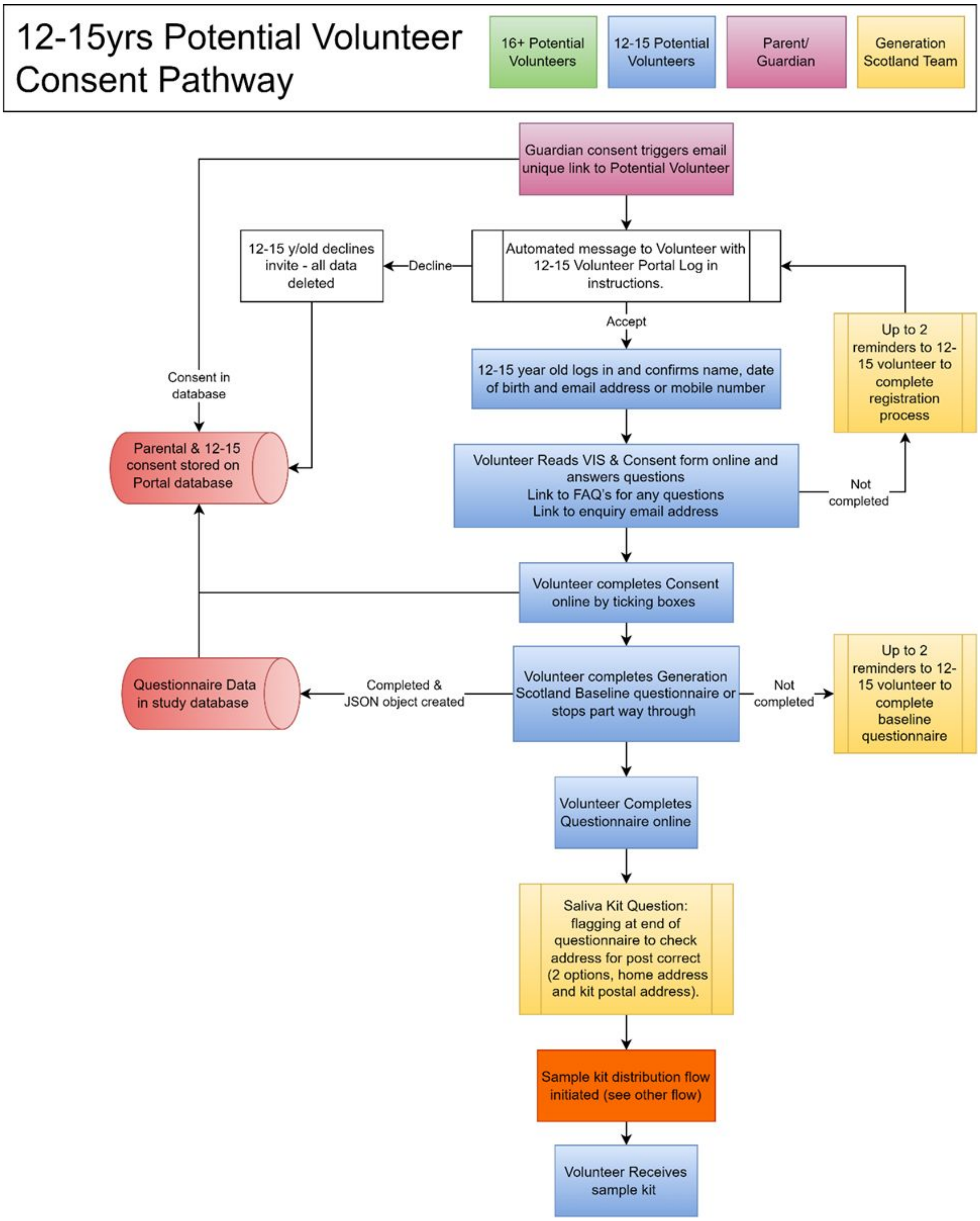

Figure.S2 12 – 15 years old potential volunteer consent pathway

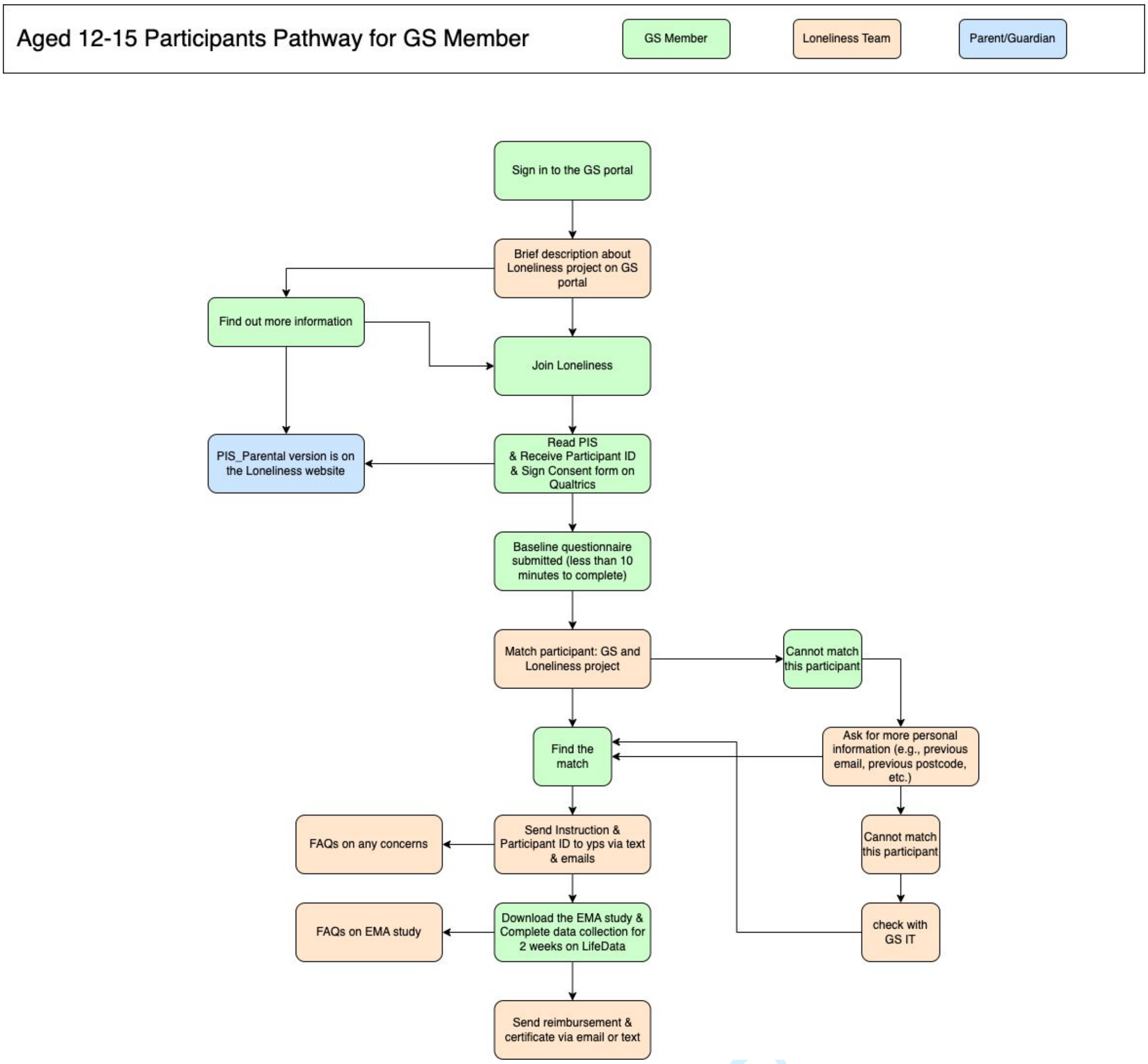

Figure.S3 Study Flow for young people who already are GS members

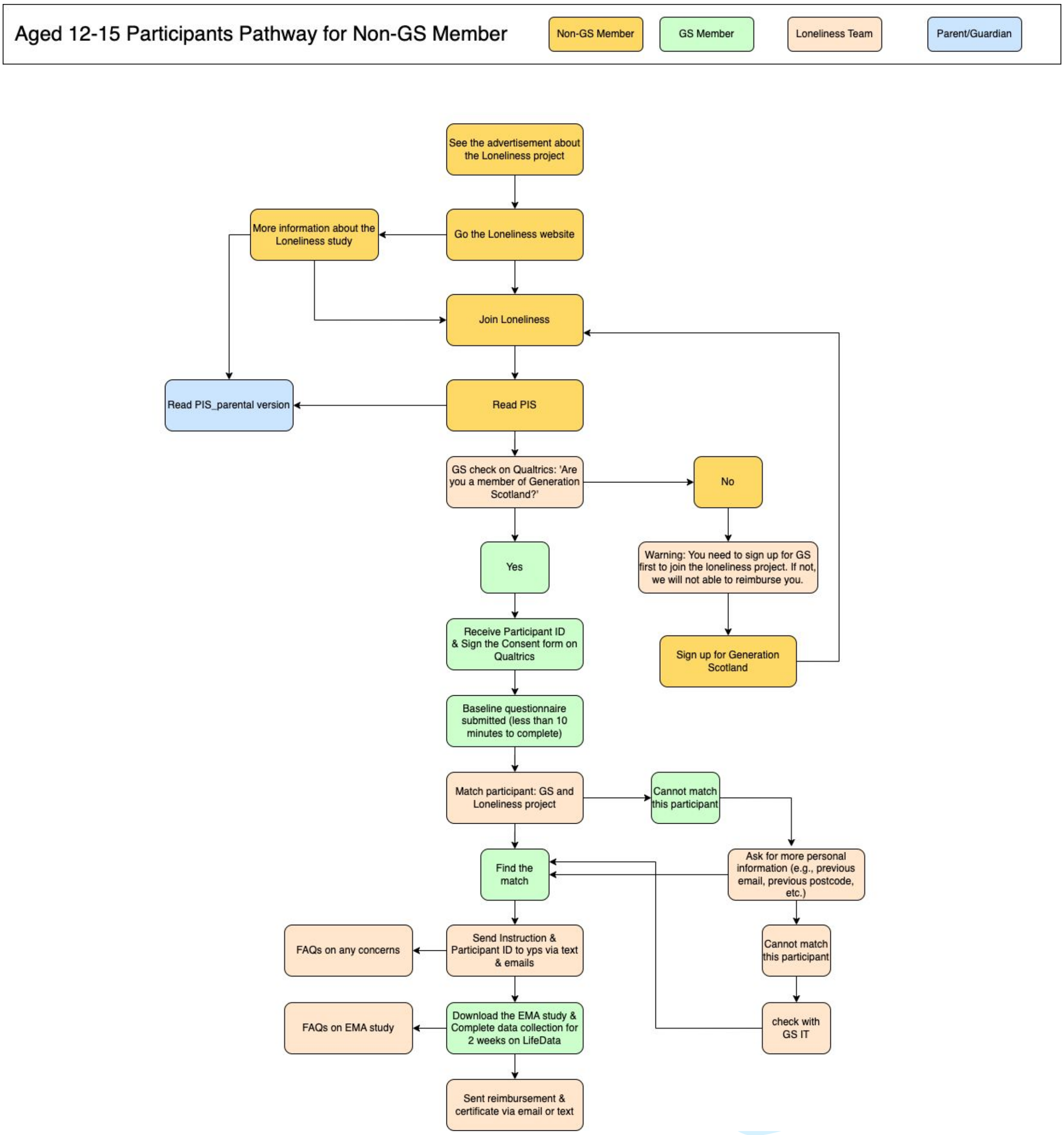

Figure.S4 Study Flow for young people who are not members of GS

Table S1. YPAG Involvement

	Characteristics	Methods	Content
Meeting 1<br>(4th April, 2023)	9 YP (girls: 5; boys: 4)<br>Mean age: 13.7y<br>Range: 11y – 17y	Online - Zoom	- What is a YPAG and why are we creating one (led by staff)<br>- Hopes for the YPAG (from YP)<br>- What Loneliness meant to you (from YP)<br>- Reimbursement approach (led by staff)<br>- Individuals task for next time (talk to your friends/family/teachers about what loneliness means to them)
Meeting 2<br>(9th May, 2023)	6 YP (girls: 4; boys: 2)<br>Mean age: 12.8y)<br>Range: 12y – 14y	Online – Zoom	- Review of previous meeting (led by staff)<br>- Presentations and discussion (from YP – what loneliness means to people)<br>- Introduce different types of loneliness and measures of loneliness (led by staff)<br>- Shared where you have your social interaction (from YP)<br>- Shared which types of social interaction you preferred (from YP)<br>- Tasks for next time (what loneliness means to people? Or where young people interact with others?)
Meeting 3<br>(30th May, 2023)	6 YP (girls: 4; boys: 2)<br>Mean age: 13.6y<br>Range: 12y – 15y	Online – Zoom	- Recap (led by staff)<br>- Overall aims of the loneliness project (led by staff)<br>- Group shared their presentation (from YP)<br>- Discussions around whether things have changed since and during lockdowns (led by staff and YP discussion)<br>- Brief overview of Generation Scotland (led by staff)<br>- Social Interaction and Loneliness Introduction (led by staff)
Meeting 4<br>(5th July, 2023)	6 YP (girls: 4; boys: 2)<br>Mean age: 13.6y<br>Range: 12y – 15y	Online – Zoom	- Introduction to ecological momentary assessment (led by staff)<br>- Discussion on EMA use (led by staff and YP discussion)<br>◊ Phones at school<br>◊ Length and frequency of prompts and questions<br>◊ Barriers

For peer review only

			◊ Motivation<br>- Questionnaire and EMA feedback (from YP)
Meeting 5<br>(25th July, 2023)	3 YP (girls: 1; boys: 2)<br>Mean age: 13.3y<br>Range: 12y – 14y	Online – Zoom	- Recap (led by staff)<br>- Questionnaire results (led by staff and YP discussion)<br>◊ Number of prompts<br>◊ School hours<br>- Feedback on EMA design (from YP)
Meeting 6<br>(19th August, 2023)	7 YP (girls: 4; boys: 3)<br>Mean age: 14.2<br>Range: 11y – 17y	In-person – University Library	- Recap (led by staff)<br>- Piloting EMA (from YP)<br>- Feedback on EMA app (from YP)<br>- Feedback on baseline questionnaire (from YP)<br>- Loneliness artwork creation (from YP)<br>- Short video for social media platform – why joins this project (from YP)

Notes. YP = Young Person; YPAG = Young Person Advisory Group; EMA = Ecological Momentary Assessment.

For peer review only

Total Prompt: 4 (daily) * 14 (days) = 56 prompts
Total items:
In-person Trigger: 22
Online Trigger: 24
No Interaction Trigger: 19

Start-up Block
Label: Informed Consent

You have already received information about this study and have agreed to take part. Please check the box below to confirm that this is the case.

If you have any questions, please email us at genscot_teens@ed.ad.uk, or text or call us at 07385 008242.

☐ I completed the consent form to take part in this study.

Label: Participant ID

Please enter your **Participant ID**

(The one you received from the previous survey)

Notification Initiated Session
[Block 1] Introduction – with graphic (56 prompt)

> Tell me about your most significant **Online** social interaction in the last hour. This can be any social interaction, big or small, positive or negative.
> Tell me about your most significant **In-person** social interaction in the last hour. This can be any social interaction, big or small, positive or negative.

[Block 2] Part 1: Interaction (56 prompts)

> Have you had any ONLINE social interaction in the last hour?
- Yes (Branch: 'Online')
- No, I didn't have online social interaction.
- No, but I had in-person social interaction. (Branch: 'In-person')

> Have you had any In-person social interaction in the last hour?
- Yes (Branch: 'In-person')
- No, I didn't have in-person social interaction
- No, but I had online social interaction. (Branch: 'Online')

Branch: 'Online':
Social platform (1 items – Multiple Choice):

> Please choose the App the interaction took place on…
- WhatsApp / SMS / Emails
- TikTok
- Snapchat
- YouTube

For peer review only

- Instagram
- Twitter (i.e., X)
- BeReal
- Discord
- Facebook
- Roblox
- Pinterest
- Amino
- Reddit
- Others (please specific)

Partner (1 items – Multiple Choice):

> Who was the main person this interaction was with?
- Friends
- Other Peers
- Family
- Known adults
- Someone not known to you
- Others (please specific)

Feeling (2 items – Multiple Choice)

> In relation to the person/people you had the interaction with… To what extent did you feel:
- Strongly rejected
- Rejected
- Neither rejected nor accepted
- Accepted
- Strongly accepted
- Not applicable

> In relations to the person/people you had the interaction with… To what extent did you feel:
- Strongly unpleasant
- Unpleasant
- Neither unpleasant nor pleasant
- Pleasant
- Strongly pleasant
- Not applicable

Branch: 'In-person':

Partner (1 items – Multiple Choice):

> Who was the main person this interaction was with?
- Friends
- Other Peers
- Family
- Known adults
- Someone not known to you
- Others (please specific)

Feeling (2 items – Multiple Choice)

> In relation to the person/people you had the interaction with…
To what extent did you feel:
- Strongly rejected
- Rejected
- Neither rejected nor accepted
- Accepted
- Strongly accepted
- Not applicable

> In relations to the person/people you had the interaction with…
To what extent did you feel:
- Strongly unpleasant
- Unpleasant
- Neither unpleasant nor pleasant
- Pleasant
- Strongly pleasant
- Not applicable

[Block 3] Introduction_Positive (~30 random character images)

> In this section, we will ask you questions related to **POSITIVE** emotions. Tell us, in the last hour, did you feel…

[Block 4] Positive Emotions (5 items – Multiple Choice)

> Happy
- Not at all
- Somewhat
- Moderately
- Very
- Extremely
- Not applicable

> Excited
- Not at all
- Somewhat
- Moderately
- Very
- Extremely
- Not applicable

> Enthusiastic
- Not at all
- Somewhat
- Moderately
- Very
- Extremely
- Not applicable

> Sense of belonging

For peer review only

- Not at all
- Somewhat
- Moderately
- Very
- Extremely
- Not applicable

> Determined
- Not at all
- Somewhat
- Moderately
- Very
- Extremely
- Not applicable

[Block 5] Introduction_Negative (~30 random character images)

> In this section, we will ask you questions related to **NEGATIVE** emotions. Tell us, in the last hour, did you feel…

[Block 6] Negative Emotions (5 items – Multiple Choice)

> Ashamed
- Not at all
- Somewhat
- Moderately
- Very
- Extremely
- Not applicable

> Nervous
- Not at all
- Somewhat
- Moderately
- Very
- Extremely
- Not applicable

> Distressed
- Not at all
- Somewhat
- Moderately
- Very
- Extremely
- Not applicable

> Upset
- Not at all
- Somewhat
- Moderately
- Very
- Extremely
- Not applicable

For peer review only

> Teased
  - Not at all
  - Somewhat
  - Moderately
  - Very
  - Extremely
  - Not applicable

[Block 7] Introduction_Loneliness (~30 random character images)

> In this section, we will ask you questions related to **LONELINESS**. Tell us, in the last hour, did you feel…

[Block 8] Loneliness (3 items – Multiple Choice)

> Lack of companionship
  - Strongly agree
  - Agree
  - Neither agree nor disagree
  - Strongly disagree
  - Disagree
  - Not applicable

> Left out
  - Strongly agree
  - Agree
  - Neither agree nor disagree
  - Strongly disagree
  - Disagree
  - Not applicable

> Isolated
  - Strongly agree
  - Agree
  - Neither agree nor disagree
  - Strongly disagree
  - Disagree
  - Not applicable

[Block 9] Anxiety (2 items – Multiple Choice)

> How anxious did you feel in the last hour?
  - Not at all
  - Somewhat
  - Moderately
  - Very
  - Extremely
  - Not applicable

> How worried did you feel in the last hour?
  - Not at all
  - Somewhat
  - Moderately
  - Very

For peer review only

- Extremely
- Not applicable

[Block 10] Depression (2 items – Multiple Choice)

> How low or depressed did you feel in the last hour?

- Not at all
- Somewhat
- Moderately
- Very
- Extremely
- Not applicable

> How hopeless did you feel in the last hour?

- Not at all
- Somewhat
- Moderately
- Very
- Extremely
- Not applicable

[Block 11] Unpleasant/Pleasant Events (1 item – Multiple Choice)

> In the last hour has anything happened that has affected your emotions positively or negatively that is not linked to social interaction (e.g., want to cinema, exam, missed the bus)

- Yes – Positive (Branch: 'Yes')
- Yes – Negative
- No

Branch Yes:

> How have these events affected your emotions?

- Not at all
- Somewhat
- Moderately
- Very
- Extremely
- Not applicable

[Block 12] Fun Stuff (~54 random images)

[Block 13] Reminder

For peer review only

BMJ Open

Loneliness in the Digital World: protocol for a co-produced Ecological Momentary Assessment study in adolescents

Journal:	*BMJ Open*
Manuscript ID	bmjopen-2024-087374.R1
Article Type:	Protocol
Date Submitted by the Author:	19-May-2024
Complete List of Authors:	Xiao, Zhuoni; University of Edinburgh Western General Hospital, Centre for Genomic and Experimental Medicine<br>Robertson, Sarah; University of Edinburgh Western General Hospital<br>Long, Emily; University of Glasgow,<br>Flaig, Robin; University of Edinburgh Western General Hospital<br>Kirby, Liz; University of Edinburgh Western General Hospital<br>Romaniuk, Liana; University of Edinburgh, Division of Psychiatry<br>Murray, Aja; The University of Edinburgh, Department of Psychology<br>Whalley, Heather; University of Edinburgh, Centre for Clinical Brain Sciences Centre for Genomic and Experimental Medicine
**Primary Subject Heading**:	Mental health
Secondary Subject Heading:	Research methods
Keywords:	Child & adolescent psychiatry < PSYCHIATRY, Psychometrics, MENTAL HEALTH

SCHOLARONE™ Manuscripts

Loneliness in the Digital World: protocol for a co-produced Ecological Momentary Assessment study in adolescents

Zhuoni Xiao[1,2]*, Sarah Robertson[1,2], Emily Long[3], Robin Flaig[1], Liz Kirby[1], Liana Romaniuk[2], Aja Murray[2,4Ω], Heather Whalley[1,2Ω]

[1] Generation Scotland, Centre for Genomic and Experimental Medicine, Institute of Genetics and Cancer, University of Edinburgh

[2] Division of Psychiatry, Centre for Clinical Brain Sciences, University of Edinburgh

[3] School of Health and Wellbeing, University of Glasgow

[4] Department of Psychology, University of Edinburgh

*Corresponding author at: Western General Hospital, Crewe Road, Edinburgh

email: zxiao@ed.ac.uk

[Ω] Authors contributed equally

For peer review only

Abstract

Introduction: Loneliness has been identified as an important public health issue, peaking during adolescence. Previous research has suggested that social interaction is a key factor in loneliness, and positive social interaction can act as a protective factor against loneliness. However, it is unclear whether there are differing impacts of in-person and online social interaction on adolescents' loneliness and mental health. Ecological Momentary Assessment (EMA) designs are ideally suited to better understanding these associations.

Method and Analysis: In the 'Loneliness in the Digital World' study, we will use a co-developed EMA design to capture daily social interactions, loneliness, and mental health such as positive and negative emotions, depression, and anxiety in approximately 200 adolescents aged 12 – 15 years. We will combine this with comprehensive information gathered from online surveys. Analysing the data using techniques such as dynamic structural equation modelling, we will examine, among other research questions, the associations between online and in-person social interaction and feelings of loneliness. The results can help inform interventions to support adolescents with high levels of loneliness and poor mental health.

Ethics and Dissemination: We received the ethics approval for the data collection from The Academic and Clinical Central Office for Research and Development (ACCORD), followed by the College of Medicine and Veterinary Medicine Ethics panel at University of Edinburgh, and finally reviewed by East of Scotland Research Ethics Service. The results will be disseminated through journal publications, conferences, and seminar presentations and to relevant stakeholders such as teachers.

For peer review only

Strengthens and Limitations of this study

- ◊ This study will use an Ecological Momentary Assessment (EMA) design to collect near real-time data, capturing young people's social interaction experiences, loneliness, and moods in their natural environments which minimizes recall bias and provides a more accurate reflection of daily life.
- ◊ This study used a co-production method involving formulating research questions, determining measurements, establishing an acceptable frequency of prompts for young people, creating appealing design of the app prompts, and deciding the optimal timing for prompt delivery, providing young people insight into the project.
- ◊ One potential limitation is that young people who agree to engage in EMA study may differ systematically from those who decline, including due to our instruction to consider not taking part if the topic is likely to trigger negative reactions.

For peer review only

Introduction

Loneliness has significant long-term consequences for physical and mental health (1). Higher levels of loneliness predict physical health conditions, poorer mental health, more frequent sleep disturbances, as well as greater cognitive difficulties later in life (2,3). Loneliness is especially salient for young people as they strive for independence and increasingly prioritize peer over familial relationships (4). Previous research has suggested that social interaction is a key factor in loneliness (5). During adolescence young people become particularly sensitive to social rejection (6,7), placing them at a higher risk of loneliness (8). The COVID-19 pandemic has further exacerbated this issue, with increased loneliness observed among younger ages since 2020 (9,10).

Adolescence is a critical period that encompasses puberty, significant brain development (6), and an increased vulnerability for internalising problems such as social anxiety (11). A previous meta-analysis conducted by Solmi et al. (12) found that across 192 studies the peak age of onset of any mental disorders was age 14.5 years. However, many researchers focus on participants over 16 years, who are easier to recruit and consent. Hence, it is important to thoroughly investigate the understudied younger adolescent population. In this study, we will leverage our established connection with Generation Scotland to facilitate the recruitment of participants from this less-explored demographic. This strategic collaboration enables us to gain valuable insights into the loneliness and mental health of age group that has been previously overlooked relative to other age groups in this area of research.

An important consideration in studying loneliness is that young people are spending more time engaging with electronic devices and online interaction compared to previous generations where social interaction were more 'in-person'. Evidence on whether the amount of time young people spend online affects mental health is mixed. Indeed, there may be both

For peer review only - http://bmjopen.bmj.com/site/about/guidelines.xhtml

risks and benefits (13). Some studies have suggested that the increase in social media use is linked to increase in loneliness and mental illness (14); however, others have suggested no meaningful associations (15). This conflicting literature is likely due to limited understanding of how young people interact online, as well as limitations in the way that these interactions are measured. The impact of the digital world on young people's mental health remains unclear, necessitating nuanced research on online social interactions (16).

Smartphone and social media usage are pervasive among adolescents, comprising the majority of their total screen time (17). In the UK, a substantial percentage (98%) of adolescents aged 12 - 15 have smartphones, and it is unsurprising that these devices were their most-used devices for accessing the internet (96%). Additionally, 93% of adolescents actively engage in social media, with WhatsApp being the most-used app among 12 – 15 (80%), followed by Snapchat (62%), Instagram (46%), and TikTok (44%) (18). This is consistent with trends observed in other Western populations (19). Samples in the US and UK (2021-2022) estimate that the time adolescents spend on smartphones and social media ranges between one and a half hours to eight and a half hours per day (20) with most adolescents spending between one and three hours per day (18). Social media platforms provide nearly constant opportunities for individuals to connect and interact with others, irrespective of the time of day or geographical location (21). Nonetheless, it remains unclear whether online and offline social interaction have distinct effects on the levels of loneliness and mental health among adolescents.

The high levels of engagement of adolescents with digital technology can also be leveraged to gather high temporal resolution, ecologically valid research data to begin to address these questions. EMA has gained significant interest since the 2000s, with a notable increase in studies published annually (22). Encouragingly, qualitative data suggests that young people find EMA easy to engage with and report that it enhances their understanding of their own behaviours, feelings, and experiences (23–26). Furthermore, a comprehensive review by

For peer review only

Wen et al. (27)examining compliance to EMA protocols in adolescents and children indicated that, across 42 studies, the weighted compliance rate stood at 78.3%. Moreover, there was no significant difference in compliance rates between studies involving clinical (76.9%) and non-clinical (79.2%) populations. Notably, among nonclinical studies, those prompting participants 2-3 times daily demonstrated a higher average compliance rate (91.7%) compared to studies with more frequent prompts (4-5 times: 77.4%; 6+: 75%). The reported compliance rates remained consistent across different durations of the EMA period in both clinical and nonclinical settings. Therefore, EMA holds promise as a method for studying young people's mental health, particularly in relation to their online world.

A crucial aspect of the study will involve developing best practice guidance on conducting Ecological Momentary Assessment in collaboration with the Young Person's Advisory Group (YPAG). YPAGs, conceptualised to enhance the acceptability and feasibility of research studies, play a crucial role in considering participants' perspectives and amplifying the relevance and impact of findings (28). The increasing prioritisation of young people's voice, underscored by the UN Convention on the Rights of the Child, is now integrated into research guidance (29). There is a growing emphasis on involving young people as 'co-actors' in health research. Engaging in research brings various benefits for young people (30) as well, including meaningful impact, the opportunity to be part of a supportive community, chances for learning and growth, and increased opportunities overall. Consequently, involving YPAGs can be considered imperative for maximising the relevance and benefits of a research project in the realm of health research.

This study will explore how various types of social interaction, both positive and negative, impact state loneliness (i.e., a more temporary and situational experience of loneliness, which may be influenced by specific events or circumstances (31) and mental health in adolescents. We will use an EMA design to assess offline/online social acceptance and

For peer review only

rejection in young people and examine how different types of social interaction affect state loneliness, emotions, and broader well-being.

Core research questions are:

- ◊ Does online social interaction and/or in-person social interaction predict later loneliness and mental health?
- ◊ Do online social interaction and in-person social interaction have the same associations to loneliness and mental health?
- ◊ Are there reciprocal relations among social interaction, loneliness, and mental health?
- ◊ Does the time of the day moderate these links?

The latter research question was suggested by the YPAG.

Exploratory analyses can be used to answer a range of additional questions such as exploration of moderation of the link between types of social interaction and loneliness by various other traits of young people. We will also develop best-practice standards for EMA research in young people, as well as validate the psychometric properties of EMA measurement tools.

Method and Analysis

Procedure

There are two phases in the current project – Phase 1: Young Person Advisory Group (YPAG) and Phase 2: full-scale study of 200 adolescents. Phase 1 is focused on conducting Patient and Public Involvement and Engagement (PPIE) work with the YPAG to co-produce a detailed protocol for the main EMA study. In Phase 2 we will launch the full EMA study, based on consultation with the YPAG.

Participants

For peer review only

Participants are expected to include approximately 200 adolescents aged 12 – 15 years from Scotland. Participants who take part in the current project will be sampled from the Generation Scotland (GS) study (32). GS is the largest family health study in Scotland and follows the health and well-being of multiple generations of families over time. Over 32,000 volunteers have already joined the study and helped research into cancer, diabetes, mental health, and other conditions (32). The current study is a new study embedded within GS. When young people join GS, their parents, or guardians have confirmed that they believe their young person has capacity to understand what is required to enrol in this study, and that this may involve follow-up studies approved by GS (See *Figure.S1* and *Figure.S2* for pathway for joining Generation Scotland in Supplementary Material A). The data gathered will be linked to participants' data from the main GS study. Participants will be offered a £20 e-voucher as compensation dependent on their response rates, as informed by the YPAG consultation processes. (See *Figure.S3* and *Figure.S4* for study flow in Supplementary Material A).

Inclusion and Exclusion criteria

Inclusion criteria for the young people will be: (1) aged 12 – 15 years old; (2) resident in Scotland; (3) Fluent in English; (4) Member of GS; (5) Able and willing to install LifeData (EMA data collection) App on their phone; if young people do not have access to a smartphone, our research team will provide one; (6) Ability to provide informed consent, and ability to independently complete online surveys and EMA protocols.

Since this study has the goal of encompassing a diverse population with various backgrounds, we have attempted to remove barriers to participation so that we can understand the broader impacts of social interaction on feelings of loneliness. Therefore, there will be no explicit exclusion criteria applied. However, given that some of the questions within the study (included aspects of social interaction, peer relations, and mood-related issues) have a small

For peer review only

risk of triggering mental health crises, we highlight in the participant informant that young people who have experienced or are currently experiencing negative social interactions should carefully consider whether they wish to participate in the study.

Patient and Public Involvement and Engagement (PPIE)

Ten young people aged 12-15yrs from diverse backgrounds and locations across Scotland were recruited via different platforms. The opportunity was shared via Young Scot and YouthLink e-briefings, LGBT Youth Scotland Discord channel and existing youth work partners, including #iwill Scotland, RespectMe, Passion4Fusion, Glasgow Disability Alliance, Glasgow Young Movers, Glasgow Youth Work Network, Intergenerations Working Together, See Me Scotland and Salvesen Mindroom Centre. The School Health and Wellbeing Improvement Research Network (SHINE) promoted the opportunity to schools via their newsletter and we delivered a presentation at the SHINE annual conference. In addition, GS promoted the YPAG through posters which were shared on social media platforms including those most popular with young people, such as TikTok and Instagram. Six YPAG meetings were held, and major changes were implemented based on their input to the protocol. For instance, YPAGs suggested that in-person social interactions are more salient than online social interaction; therefore, instead of asking the young people to recall their most impactful social interaction in the past hour, we now randomly ask them to recall their most impactful in-person or online social interaction in the past hour. Additionally, changes were made based on their input, such as reducing the length of the surveys, suggesting the time for each prompt, adding a progress bar for EMA surveys, incorporating a 'not applicable' option for all items, and embedding more fun and educational elements into the study. These elements include providing cartoon characters for each introduction, offering 'Fun Images' and 'Brain Facts' at the end of each completed EMA survey. As per the recommendations by Sellars et al. (33) regarding consistent reporting of YPAG involvement, YPAG characteristics, frequency of

For peer review only

engagement, methods and extent of involvement, parental engagement, and recognition of involvement are presented in Table S1 of the Supplementary Material A.

Data Collection

Data collection will begin in April 2024, and be completed in six months. *Figure.1* summarises the study flow. Informed consent will be collected from the young people online, and then they will be invited to complete an online baseline survey.

On completion of the online baseline survey, participants will initiate the EMA surveys. Once researchers confirmed they are members of GS, participants will be provided with a set of instructions for downloading the App, and offered on-boarding support via email, text, or call. Participants will use their own smartphone where possible; however, to prevent selection biases and encourage inclusivity, study-provided smartphones will be made accessible. An email helpline and phone number will also be available for participants to receive help on any issues they encounter.

EMA data collection will take place over a two-week period and involve four prompts per day at 8:00 – 9:00 am, 12:30 – 1:30 pm, 5:30 – 6:30 pm, and 10:00 – 11:00 pm. All of these will be administered via a smartphone application provided by LifeData. All measures will be designed to take less than one minute as lengthy questionnaires are linked to heightened burden and decreased compliance (34). Consistent with this, our YPAG emphasised the importance of minimising burden.

At the end of the two-week EMA period, participants will be offered an e-voucher worth up to £20, with the exact amount to be dependent on their response rates (£5 for completion of baseline questionnaire and different amounts for EMA protocol depend on completion rate - £15 for 70% completion rate; £10 for 50% completion rate; £5 for 30% completion rate; £2 for under 30% completion rate). They will also receive a participation

For peer review only

certificate. EMA surveys are provided in the online Supplementary Materials B. *Figure.2* presents the screenshots of the EMA protocol on phone.

Measures

The baseline questionnaire will be collected online via Qualtrics, and the EMA survey will be collected via the LifeData app. The measures that young people will complete over the course of the study are presented in Table S2 in the Supplementary Material A.

Statistical Procedure

Data will be analysed using Dynamic Structural Equation Modelling (DSEM) (35–37). DSEM combines multi-level structural equation, and time-series modelling, which allows an examination of individual differences in within-person dynamics such as the connections between social interaction and emotions. We will utilize a multilevel CFA model to examine the psychometric properties of a three-item loneliness measurement. Then, we will use DSEM with lag-1 autoregressive and cross-lagged effects to examine the impacts of social rejection/acceptance on itself over time, and loneliness or emotions at a later time. We will also utilize mediation within DSEM to explore whether state emotions mediate the associations between social interaction and loneliness or mental wellbeing. We will be using time-varying effects DSEM to investigate whether time of the day moderate these associations. These models will be implemented in the latest available versions of Mplus and R.

Sample Size Justification

We will use a sample size of N = 200, adolescents aged 12 to 15 years old. Power analyses are based on a Monte Carlo approach, setting a threshold of $\alpha = 0.05$ and $1- \beta = .80$. Using this approach, the sample size necessary to detect significant effects in key parameters (e.g., moderation of a cross-lagged within-person effect) based on realistic parameter estimates was explored. These realistic parameter estimates were taken from DSEM models fit to past

For peer review only - http://bmjopen.bmj.com/site/about/guidelines.xhtml

similar EMA data by Murray and team (38). We determined that an N = 100 would provide sufficient power. Therefore, a sample size of N = 200 would provide a strong buffer against attrition or missingness, as well as allow us to examine sub-groups such as by gender.

Ethics and Dissemination

We received the ethics approval for the data collection from The Academic and Clinical Central Office for Research and Development (ACCORD), followed by the College of Medicine and Veterinary Medicine Ethics panel at University of Edinburgh, and finally reviewed by East of Scotland Research Ethics Service. The results will be disseminated through journal publication, conferences, and seminar presentations.

Discussion

The current protocol describes the design for a joint traditional and EMA survey study of the daily life social interactions and their impacts of adolescents' loneliness and mental wellbeing. We will build capacity and guidance on conducting robust, youth-led research on adolescent mental health, through engagement and methodological innovation. Collaborating with young people aged 12 to 15 years, we will help contribute to best-practice standards for EMA research, psychometrically validated measurement tools, and apply cutting-edge analysis methods for these complex data. We will gather information on whether different types of social interactions have the same impacts on loneliness and mental wellbeing, whether there are gender differences in these associations, whether social rejection affects subsequent emotions and loneliness, and whether current social acceptance would help recovery from previous social rejection by reducing loneliness or improving mental wellbeing. Our findings can therefore inform better understanding of the impacts of social interaction on loneliness and illuminate poorly understood areas of adolescent mental health with implications for policy

For peer review only

and clinical practice. For example, the World Health Organisation Commission on Social Connection (2024 – 2026) aims to see loneliness be recognised and resourced as a global public health priority. Our research could provide preliminary findings for developing solutions for loneliness, as well as valid measurements for assessing loneliness as it plays out in real-time. Our findings can help inform, for example, prescribe culture (e.g., better peer relations or better online social behaviours to improve mental health and wellbeing).

Previous interventions for loneliness focus on different aspects. For instance, social skills (39–42), social interaction (43–45), emotional skills (41), enhanced social support (46–49) or psychological interventions (46,50) However, most interventions are targeted at adults, older adults, or patients' groups, rather than tailored for the needs adolescents. Findings from the current project could inform interventions tailored for adolescents and that reflect their social interaction habits such as promoting healthy online behaviours.

Our methodology has the potential to accelerate advancements in the utilization of Ecological Momentary Assessment (EMA) technique within adolescent populations in school setting. Through our research, we aim to investigate the impact of prompt timing on response rates, specifically exploring whether prompts during school hours versus after-school hours influence response rates. By providing a template for such research (which we will make openly available in the form of openly accessible study materials, including measures and analysis code), and systematically recording challenges, solutions, and advantages, this study can foster broader adoption of EMA methodologies.

Limitations

While extensive research has explored optimal design features for Ecological Momentary Assessment (EMA) in general (34,51), the relative novelty of the approach for measuring social interactions in adolescents means there is limited past research to guide key

For peer review only

methodological decisions such as measure selection, development or validation, sampling frequency and schedules, reimbursement, and strategies to promote engagement (52). There may also be difficulties in gathering information during school hours meaning that we may have high levels of missingness during the day. Finally, the sample will be drawn from the GS study. While this has the benefit of providing comprehensive measures to link to the EMA data to, the sample may be selective in the sense that they have demonstrated a motivation to engage in health research.

For peer review only

Reference:

1. Park C, Majeed A, Gill H, Tamura J, Ho RC, Mansur RB, et al. The Effect of Loneliness on Distinct Health Outcomes: A Comprehensive Review and Meta-Analysis. Psychiatry Res. 2020 Dec 1;294:113514.

2. Heinrich LM, Gullone E. The clinical significance of loneliness: A literature review. Clin Psychol Rev. 2006 Oct 1;26(6):695–718.

3. Lim MH, Eres R, Vasan S. Understanding loneliness in the twenty-first century: an update on correlates, risk factors, and potential solutions. Soc Psychiatry Psychiatr Epidemiol. 2020 Jul 1;55(7):793–810.

4. Loades ME, Chatburn E, Higson-Sweeney N, Reynolds S, Shafran R, Brigden A, et al. Rapid Systematic Review: The Impact of Social Isolation and Loneliness on the Mental Health of Children and Adolescents in the Context of COVID-19. J Am Acad Child Adolesc Psychiatry. 2020 Nov 1;59(11):1218-1239.e3.

5. Osborn T, Weatherburn P, French RS. Interventions to address loneliness and social isolation in young people: A systematic review of the evidence on acceptability and effectiveness. J Adolesc. 2021 Dec 1;93:53–79.

6. Fuhrmann D, Knoll LJ, Blakemore SJ. Adolescence as a Sensitive Period of Brain Development. Trends Cogn Sci. 2015 Oct 1;19(10):558–66.

7. Sebastian CL, Tan GCY, Roiser JP, Viding E, Dumontheil I, Blakemore SJ. Developmental influences on the neural bases of responses to social rejection: Implications of social neuroscience for education. NeuroImage. 2011 Aug 1;57(3):686–94.

8. Pitman A, Mann F, Johnson S. Advancing our understanding of loneliness and mental health problems in young people. Lancet Psychiatry. 2018 Dec 1;5(12):955–6.

9. Qualter P, Brown SL, Rotenberg KJ, Vanhalst J, Harris RA, Goossens L, et al. Trajectories of loneliness during childhood and adolescence: Predictors and health outcomes. J Adolesc. 2013 Dec 1;36(6):1283–93.

10. van Tilburg TG, Steinmetz S, Stolte E, van der Roest H, de Vries DH. Loneliness and Mental Health During the COVID-19 Pandemic: A Study Among Dutch Older Adults. J Gerontol Ser B. 2021 Aug 13;76(7):e249–55.

11. Ronald M R, Ella L O, Carly J J, Miriam K F, Jasmine F, Natasha R M, et al. Adolescent development and risk for the onset of social-emotional disorders: A review and conceptual model. Behav Res Ther. 2019 Dec 1;123:103501.

12. Solmi M, Radua J, Olivola M, Croce E, Soardo L, Salazar de Pablo G, et al. Age at onset of mental disorders worldwide: large-scale meta-analysis of 192 epidemiological studies. Mol Psychiatry. 2022 Jan;27(1):281–95.

13. Kardefelt-Winther D, Rees G, Livingstone S. Contextualising the link between adolescents' use of digital technology and their mental health: a multi-country study of time spent online and life satisfaction. J Child Psychol Psychiatry. 2020 Aug 1;61(8):875–89.

14. Twenge JM, Spitzberg BH, Campbell WK. Less in-person social interaction with peers among U.S. adolescents in the 21st century and links to loneliness. J Soc Pers Relatsh [Internet]. 2019 Mar 19 [cited 2024 May 14]; Available from: https://journals.sagepub.com/doi/full/10.1177/0265407519836170?casa_token=REpCpE6jK6sAAAAA%3ASV691MeSDC8LUxfpM20l-K92lrsQNFq2vuXr4Aim_cCGLE6wYLPEUYHMTyKKqEveTDV528hDYTkO

15. Jensen M, George MJ, Russell MR, Odgers CL. Young Adolescents' Digital Technology Use and Mental Health Symptoms: Little Evidence of Longitudinal or Daily Linkages. Clin Psychol Sci [Internet]. 2019 Aug 20 [cited 2024 May 14]; Available from: https://journals.sagepub.com/doi/full/10.1177/2167702619859336?casa_token=YSK-vlqkmj0AAAAA%3AlK3lTJghTjc6Y-dgUY8ivt7VOzaDZWFWkI3PRJPLJ2q0-zbnP6Jft3r5k4rIwkUX0W_IVAcf698R

16. Odgers CL, Jensen MR. Annual Research Review: Adolescent mental health in the digital age: facts, fears, and future directions. J Child Psychol Psychiatry. 2020 Mar 1;61(3):336–48.

17. U.K. O. Children and parents: media use and attitudes report 2022. 2022;

18. U.K O. Children and Parents: Media Use and Attitudes 2023. 2023;

19. Orben A. Teenagers, screens and social media: a narrative review of reviews and key studies. Soc Psychiatry Psychiatr Epidemiol. 2020 Apr 1;55(4):407–14.

20. Kontostoli E, Jones AP, Pearson N, Foley L, Biddle SJH, Atkin AJ. The Association of Contemporary Screen Behaviours with Physical Activity, Sedentary Behaviour and Sleep in Adolescents: a Cross-sectional Analysis of the Millennium Cohort Study. Int J Behav Med. 2023 Feb 1;30(1):122–32.

For peer review only

21. Naslund JA, Bondre A, Torous J, Aschbrenner KA. Social Media and Mental Health: Benefits, Risks, and Opportunities for Research and Practice. J Technol Behav Sci. 2020 Sep 1;5(3):245–57.

22. Wrzus C, Neubauer AB. Ecological Momentary Assessment: A Meta-Analysis on Designs, Samples, and Compliance Across Research Fields. Assessment [Internet]. 2022 Jan 11 [cited 2024 May 14]; Available from: https://journals.sagepub.com/doi/full/10.1177/10731911211067538

23. Acorda D, Businelle M, Maria DS. Perceived Impacts, Acceptability, and Recommendations for Ecological Momentary Assessment Among Youth Experiencing Homelessness: Qualitative Study. JMIR Form Res. 2021 Apr 6;5(4):e21638.

24. Heron KE, Everhart RS, McHale SM, Smyth JM. Using Mobile-Technology-Based Ecological Momentary Assessment (EMA) Methods With Youth: A Systematic Review and Recommendations. J Pediatr Psychol. 2017 Nov 1;42(10):1087–107.

25. Nap-van der Vlist MM, Houtveen J, Dalmeijer GW, Grootenhuis MA, van der Ent CK, van Grotel M, et al. Internet and smartphone-based ecological momentary assessment and personalized advice (PROfeel) in adolescents with chronic conditions: A feasibility study. Internet Interv. 2021 Sep 1;25:100395.

26. Russell MA, Gajos JM. Annual Research Review: Ecological momentary assessment studies in child psychology and psychiatry. J Child Psychol Psychiatry. 2020 Mar 1;61(3):376–94.

27. Wen CKF, Schneider S, Stone AA, Spruijt-Metz D. Compliance With Mobile Ecological Momentary Assessment Protocols in Children and Adolescents: A Systematic Review and Meta-Analysis. J Med Internet Res. 2017 Apr 26;19(4):e6641.

28. Kirby P. A Guide to Actively Involving Young People in Research:

29. U.G A. Convention on the Rights of the Child.

30. Watson R, Burgess L, Sellars E, Crooks J, McGowan R, Diffey J, et al. A qualitative study exploring the benefits of involving young people in mental health research. Health Expect. 2023 Aug 1;26(4):1491–504.

31. Motta V. Key Concept: Loneliness. Philos Psychiatry Psychol. 2021;28(1):71–81.

32. Smith BH, Campbell A, Linksted P, Fitzpatrick B, Jackson C, Kerr SM, et al. Cohort Profile: Generation Scotland: Scottish Family Health Study (GS:SFHS). The study, its participants and their potential for genetic research on health and illness. Int J Epidemiol. 2013 Jun 1;42(3):689–700.

33. Sellars E, Pavarini G, Michelson D, Creswell C, Fazel M. Young people's advisory groups in health research: scoping review and mapping of practices. Arch Dis Child. 2021 Jul 1;106(7):698–704.

34. Eisele G, Vachon H, Lafit G, Kuppens P, Houben M, Myin-Germeys I, et al. The Effects of Sampling Frequency and Questionnaire Length on Perceived Burden, Compliance, and Careless Responding in Experience Sampling Data in a Student Population. Assessment

For peer review only

[Internet]. 2020 Sep 10 [cited 2024 May 14]; Available from: https://journals.sagepub.com/doi/full/10.1177/1073191120957102?casa_token=Q3tp9WqoSiIAAAAA%3AZQsiGBVytitwhxkIcZbzv-HlbR7z40TrKBtMkRcQk00GC9KKY24Uh__faO7SO6LA6aFxSrTDWynv

35. Asparouhov T, Muthén B. Comparison of Models for the Analysis of Intensive Longitudinal Data. Struct Equ Model Multidiscip J [Internet]. 2020 Mar 3 [cited 2024 May 14]; Available from: https://www.tandfonline.com/doi/abs/10.1080/10705511.2019.1626733

36. Asparouhov T, Hamaker EL, Muthén B. Dynamic Structural Equation Models. Struct Equ Model Multidiscip J [Internet]. 2018 May 4 [cited 2024 May 14]; Available from: https://www.tandfonline.com/doi/abs/10.1080/10705511.2017.1406803

37. McNeish D, Hamaker EL. A primer on two-level dynamic structural equation models for intensive longitudinal data in Mplus. Psychol Methods. 2020 Oct;25(5):610–35.

38. Murray A, Ushakova A, Zhu X, Yang Y, Xiao Z, Brown R, et al. Predicting Participation Willingness in Ecological Momentary Assessment of General Population Health and Behavior: Machine Learning Study. J Med Internet Res. 2023 Aug 2;25(1):e41412.

39. Barry TD, Klinger LG, Lee JM, Palardy N, Gilmore T, Bodin SD. Examining the Effectiveness of an Outpatient Clinic–Based Social Skills Group for High-Functioning Children with Autism. J Autism Dev Disord. 2003 Dec 1;33(6):685–701.

40. Beidel DC, Turner SM, Morris TL. Behavioral treatment of childhood social phobia. J Consult Clin Psychol. 2000;68(6):1072–80.

41. Bostick D, Anderson R. Evaluating a Small-Group Counseling Program—A Model for Program Planning and Improvement in the Elementary Setting. Prof Sch Couns [Internet]. 2018 Feb 15 [cited 2024 May 14]; Available from: https://journals.sagepub.com/doi/abs/10.1177/2156759X0901200602?casa_token=iPOxnBuRQJEAAAAA%3AgfWPQN-pNCX-Mhs5-jGMyeEZtEVZeKYKT7BLUcRn2SAyA7lARuyzcOATTB_COKlvx5oUdOXOSO07

42. King GA, Specht JA, Schultz I, Warr-Leeper G, Redekop W, Risebrough N. Social skills training for withdrawn unpopular children with physical disabilities: A preliminary evaluation. Rehabil Psychol. 1997;42(1):47–60.

43. Battles HB, Wiener LS. STARBRIGHT World: Effects of an Electronic Network on the Social Environment of Children With Life-Threatening Illnesses. Child Health Care [Internet]. 2002 Mar 1 [cited 2024 May 14]; Available from: https://www.tandfonline.com/doi/abs/10.1207/S15326888CHC3101_4

44. Grace E, Raghavendra P, Newman L, Wood D, Connell T. Learning to use the Internet and online social media: What is the effectiveness of home-based intervention for youth with complex communication needs? Child Lang Teach Ther [Internet]. 2014 Jan 14 [cited 2024 May 14]; Available from: https://journals.sagepub.com/doi/full/10.1177/0265659013518565?casa_token=FaDqWS2upOwAAAAA%3Au_kvyCjTNYrYvocRQ95fTUxYIU_MRAqzoACbOtd0jDwZvSYeCZA9T4SAQCYK_6xa2Jas8GWXsT64

For peer review only

45. Lim MH, Rodebaugh TL, Eres R, Long KM, Penn DL, Gleeson JFM. A Pilot Digital Intervention Targeting Loneliness in Youth Mental Health. Front Psychiatry. 2019 Aug 23;10:453345.

46. Brouzos A, Vassilopoulos SP, Moschou K. Utilizing storytelling to promote emotional well-being of children with a distinct physical appearance: The case of children who wear eyeglasses. Eur J Couns Psychol [Internet]. 2016 Mar 23 [cited 2024 May 14];4(1). Available from: https://www.psycharchives.org/en/item/c064b66f-0933-4c2c-af00-d9eaed08d0a0

47. Bradley R. 'Why single me out?' Peer mentoring, autism and inclusion in mainstream secondary schools. Br J Spec Educ. 2016 Oct 1;43(3):272–88.

48. Kneer J, van Eldik A, Jansz J, Eischeid S, Usta M. With a Little Help from My Friends: Peer Coaching for Refugee Adolescents and the Role of Social Media. Media Commun. 2019 Jun 28;7(2):264–74.

49. Stewart M, Barnfather A, Magill-Evans J, Ray L, Letourneau N. Brief report: An online support intervention: Perceptions of adolescents with physical disabilities. J Adolesc. 2011 Aug 1;34(4):795–800.

50. Smith NG, Hart TA, Kidwai A, Vernon JRG, Blais M, Adam B. Results of a Pilot Study to Ameliorate Psychological and Behavioral Outcomes of Minority Stress Among Young Gay and Bisexual Men. Behav Ther. 2017 Sep 1;48(5):664–77.

51. Rintala A, Wampers M, Myin-Germeys I, Viechtbauer W. Momentary predictors of compliance in studies using the experience sampling method. Psychiatry Res. 2020 Apr 1;286:112896.

52. Murray AL, Xie T. Engaging Adolescents in Contemporary Longitudinal Health Research: Strategies for Promoting Participation and Retention. J Adolesc Health. 2024 Jan 1;74(1):9–17.

For peer review only

Authors' contributions

Conceptualisation: HW. Funding acquisition: HW. Methodology: ZX, AM and HW. Writing – original draft: ZX. Writing – review and editing: ZX, SR, EL, RF, LK, LR, AM and HW.

Funding Statement

The research describe within this report was supported by Medical Research Council (MRC) grant (Grant Ref: MR/X002608/1), and AM was additionally supported by a Wellcome Trust Career Development Grant (REF: 225364/Z/22/Z).

Acknowledgment

We are grateful to Alasdair MacKenzie, Ava Marshall, Brody Pardoe Hay, Daniel Galloway, Eva Drummond, Gareth Stubbs, Jessica Ehimen, Sharon Ehimen, Shuman Zhao, and Tyler Hill for their assistance in designing, co-producing, shaping ideas, providing valuable feedback, and piloting the project. We also appreciate the contributions of Lorna Caddick, Nan Peng, Ercan Ozdemir, and Dejla Hoxha for their assistance in piloting and offering valuable feedback on the project.

Competing Interests Statement

N/A

For peer review only

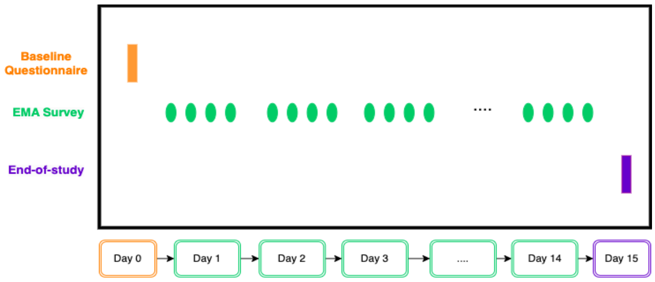

Figure.1 Summary of data collection schedule. EMA = Ecological Momentary Assessment.

For peer review only

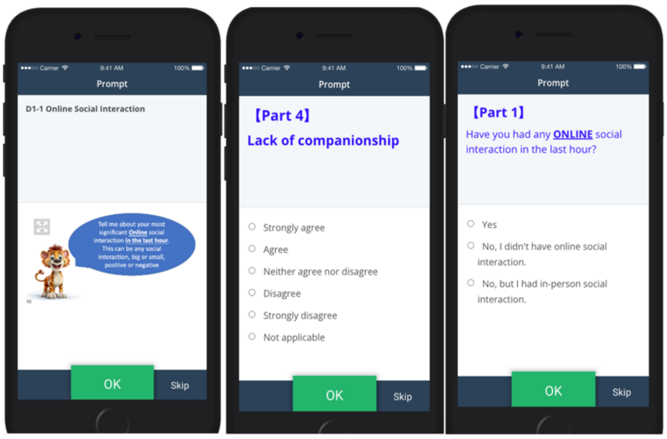

Figure.2 Screenshots of EMA protocol on phone

For peer review only

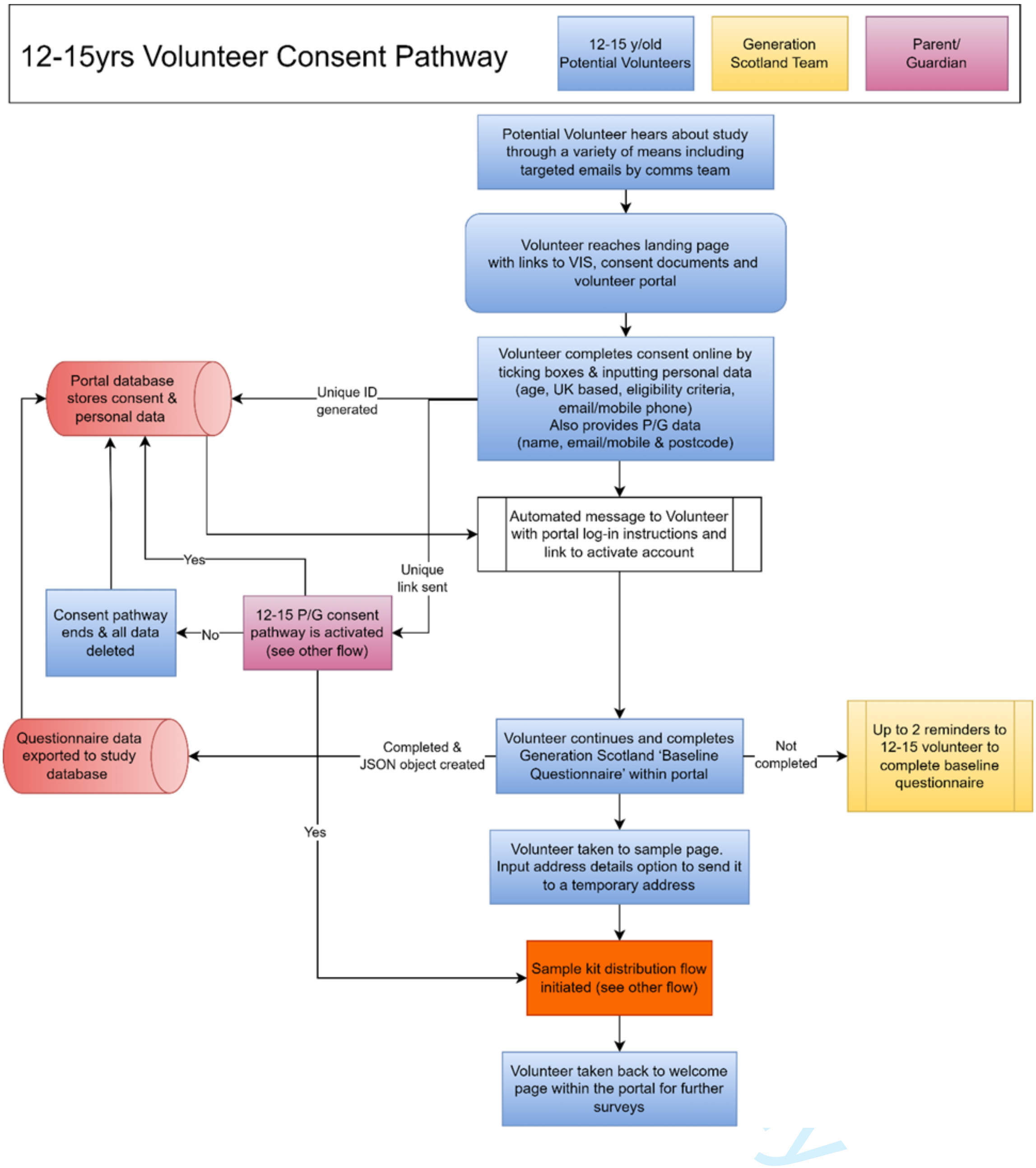

Figure.S1 12 – 15 years old volunteer consent pathway

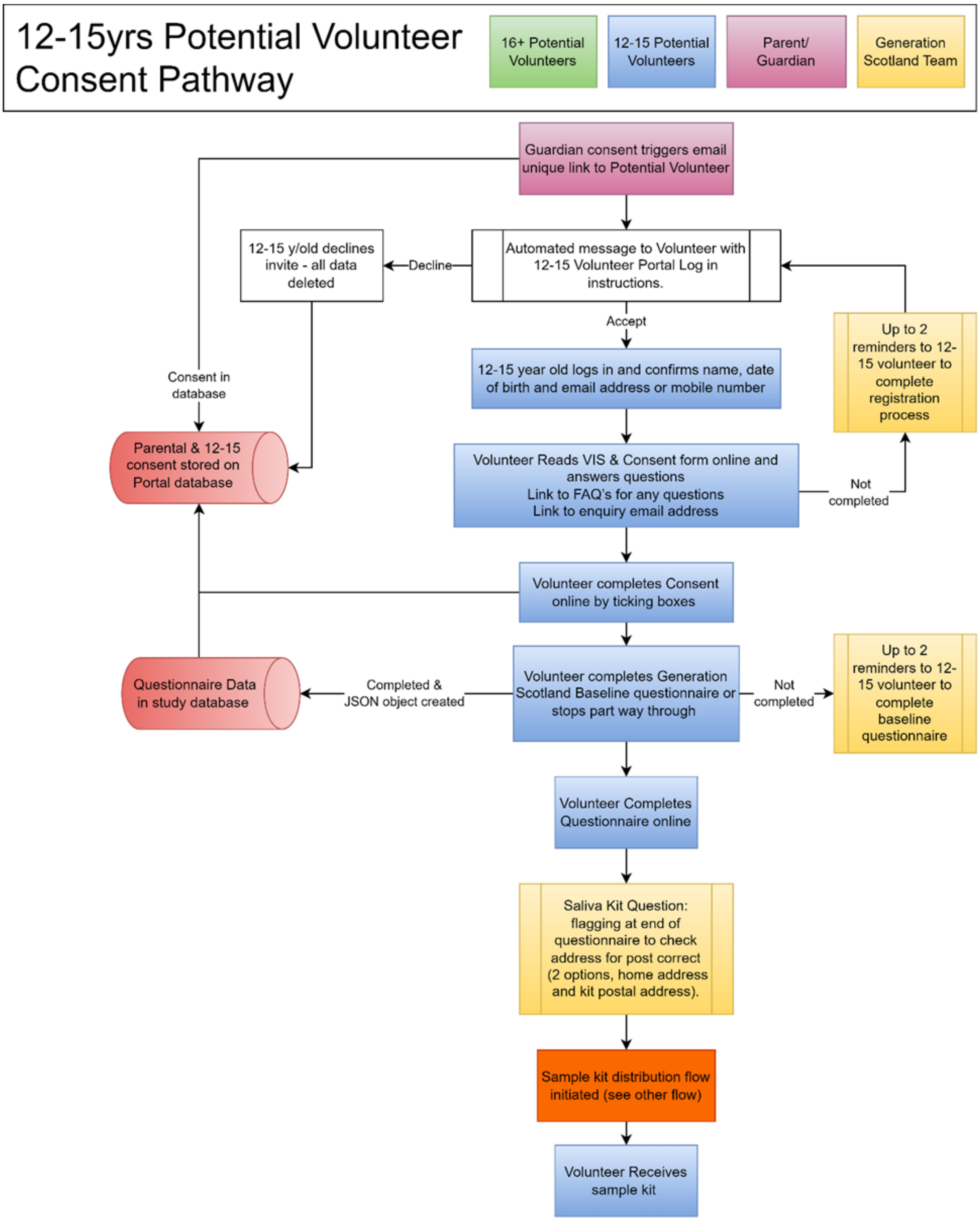

Figure.S2 12 – 15 years old potential volunteer consent pathway

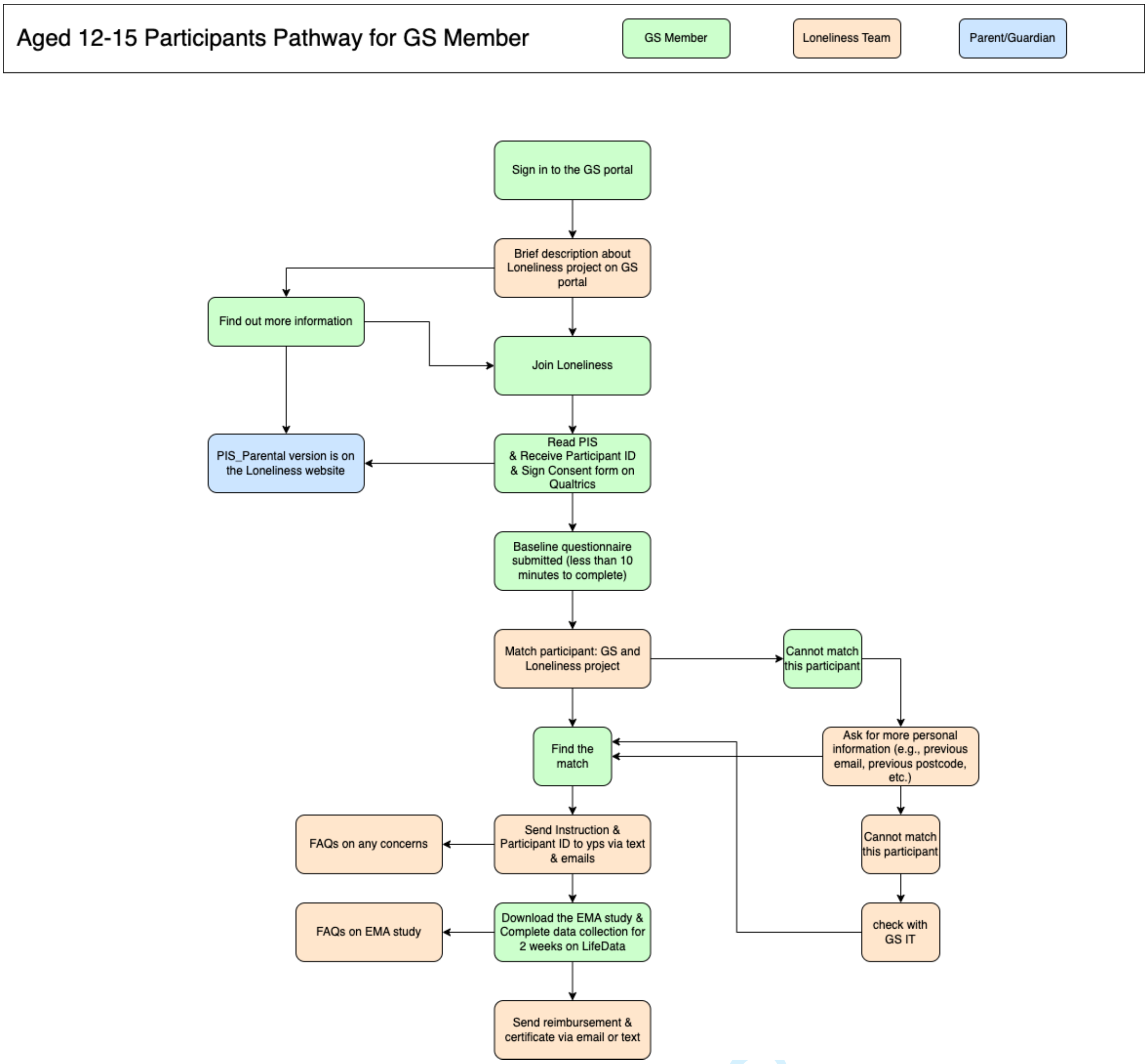

Figure.S3 Study Flow for young people who already are GS members

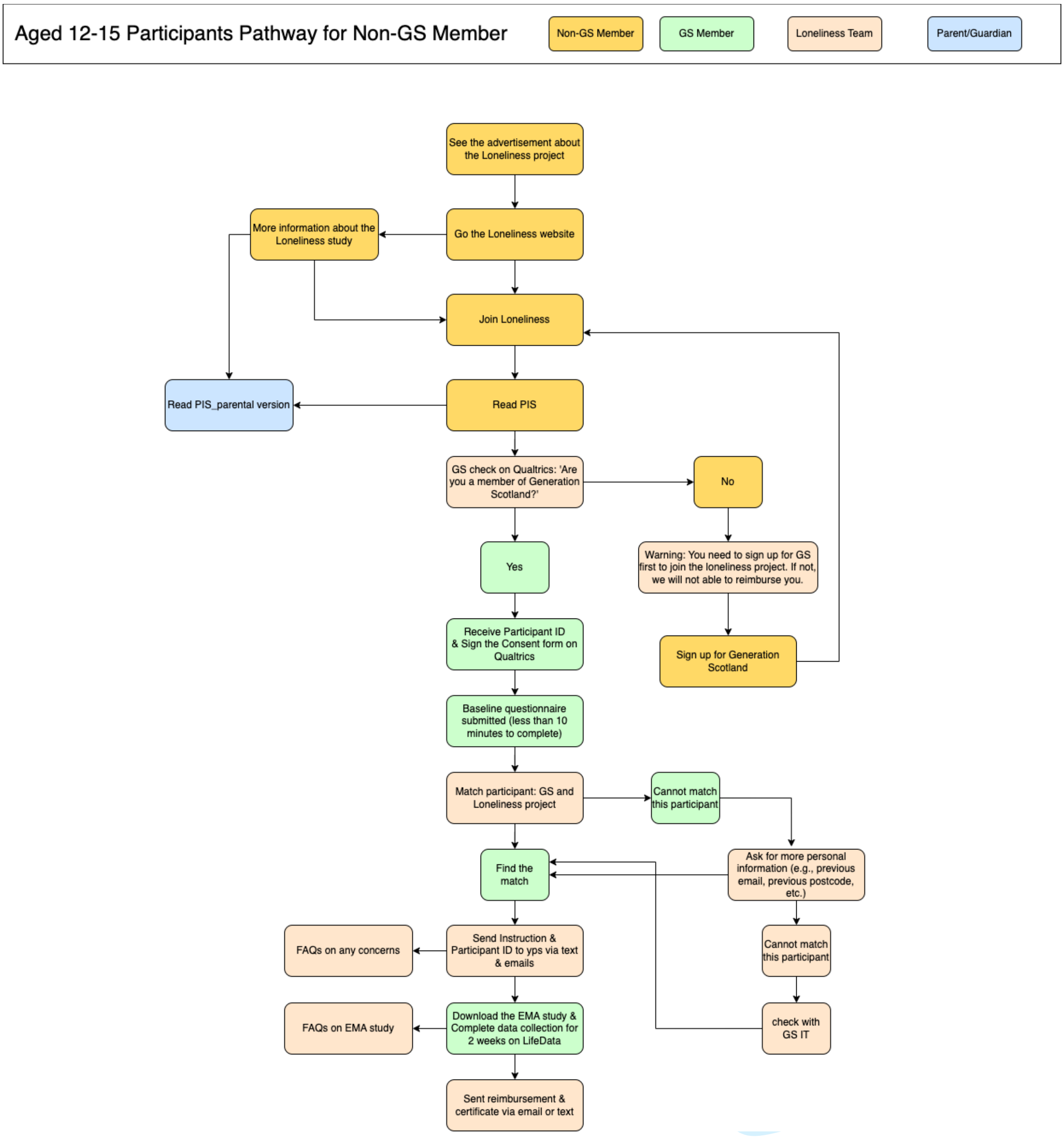

Figure.S4 Study Flow for young people who are not members of GS

Table S1. YPAG Involvement

	Characteristics	Methods	Content
Meeting 1 (4th April, 2023)	9 YP (girls: 5; boys: 4)<br>Mean age: 13.7y<br>Range: 11y – 17y	Online - Zoom	- What is a YPAG and why are we creating one (led by staff)<br>- Hopes for the YPAG (from YP)<br>- What Loneliness meant to you (from YP)<br>- Reimbursement approach (led by staff)<br>- Individuals task for next time (talk to your friends/family/teachers about what loneliness means to them)
Meeting 2 (9th May, 2023)	6 YP (girls: 4; boys: 2)<br>Mean age: 12.8y)<br>Range: 12y – 14y	Online – Zoom	- Review of previous meeting (led by staff)<br>- Presentations and discussion (from YP – what loneliness means to people)<br>- Introduce different types of loneliness and measures of loneliness (led by staff)<br>- Shared where you have your social interaction (from YP)<br>- Shared which types of social interaction you preferred (from YP)<br>- Tasks for next time (what loneliness means to people? Or where young people interact with others?)
Meeting 3 (30th May, 2023)	6 YP (girls: 4; boys: 2)<br>Mean age: 13.6y<br>Range: 12y – 15y	Online – Zoom	- Recap (led by staff)<br>- Overall aims of the loneliness project (led by staff)<br>- Group shared their presentation (from YP)<br>- Discussions around whether things have changed since and during lockdowns (led by staff and YP discussion)<br>- Brief overview of Generation Scotland (led by staff)<br>- Social Interaction and Loneliness Introduction (led by staff)
Meeting 4 (5th July, 2023)	6 YP (girls: 4; boys: 2)<br>Mean age: 13.6y<br>Range: 12y – 15y	Online – Zoom	- Introduction to ecological momentary assessment (led by staff)<br>- Discussion on EMA use (led by staff and YP discussion)<br>◊ Phones at school<br>◊ Length and frequency of prompts and questions<br>◊ Barriers<br>◊ Motivation<br>- Questionnaire and EMA feedback (from YP)

For peer review only

Meeting 5 (25th July, 2023)	3 YP (girls: 1; boys: 2)<br>Mean age: 13.3y<br>Range: 12y – 14y	Online – Zoom	- Recap (led by staff)<br>- Questionnaire results (led by staff and YP discussion)<br>◊ Number of prompts<br>◊ School hours<br>- Feedback on EMA design (from YP)
Meeting 6 (19th August, 2023)	7 YP (girls: 4; boys: 3)<br>Mean age: 14.2<br>Range: 11y – 17y	In-person – University Library	- Recap (led by staff)<br>- Piloting EMA (from YP)<br>- Feedback on EMA app (from YP)<br>- Feedback on baseline questionnaire (from YP)<br>- Loneliness artwork creation (from YP)<br>- Short video for social media platform – why joins this project (from YP)

Notes. YP = Young Person; YPAG = Young Person Advisory Group; EMA = Ecological Momentary Assessment.

For peer review only

Table S2. Measures for adolescent survey

Phase	Measures	Sources	Items, n
Baseline Questionnaire			
	Identifier	Postcode, phone number, email address	3
	Demographic	Age, sex, school transition, number of brothers and sisters, birth order, smoking in the past month, drinking in the past month, number of close friends	8
	Social Media Use	Smartphone access during weekday, smartphone access during weekend, average hours spent on social media during weekday, average hours spent on social media during weekend, how much time spent on direct message, passive message, passive in the public in the past hour, psychological aspect of social media use	10
	Attachment	Inventory of Parent and Peer Attachment (Buist et al., 2004)	11
	Depression & Anxiety	Revised Child Anxiety and Depression Scale (Chorpita et al., 2000)	16
	Personality	Ten-item Personality Inventory (Gosling, Rentfrow, & Swann Jr, 2003)	10
	Self-esteem	Rosenberg Self-Esteem Questionnaire (Rosenberg, 1965)	10

For peer review only - http://bmjopen.bmj.com/site/about/guidelines.xhtml

	Social Relationships	Social Relationships Questionnaire (Owens, & Hinshaw, 2016)	10
	Loneliness	UCLA (Russell, 1996)	20
	Perceived Social Support	Multidimensional Scale of Perceived Social Support (Zimet et al., 1988)	14
EMA Survey			
	In-person or Online Social Interaction	Have you had any online/in-person social interaction?	1
	Platform	Which social platform the interaction took place?	1
	Partner	Who was the main person this interaction was with?	1
	Feeling	Rejection/Acceptance, Pleasant/Unpleasant	2
	Positive Emotions	Adapted from Positive Affect Negative Affect Schedule expanded (Watson, & Clark, 1994)	3
	Negative Emotions	Adapted from Positive Affect Negative Affect Schedule expanded (Watson, & Clark, 1994)	3
	Loneliness	Adapted from UCLA (Russell, 1996)	3

For peer review only

Internalising Problems	Adapted from Revised Child Anxiety and Depression Scale (Chorpita et al., 2000)	6
Other Events	Any events that affected your emotions?	1

Notes. EMA = Ecological Momentary Assessment. The measurement for social media use was developed by the research team.

For peer review only

Total Prompt: 4 (daily) * 14 (days) = 56 prompts
Total items:
In-person Trigger: 22
Online Trigger: 24
No Interaction Trigger: 19

Start-up Block
Label: Informed Consent

You have already received information about this study and have agreed to take part. Please check the box below to confirm that this is the case.

If you have any questions, please email us at genscot_teens@ed.ad.uk, or text or call us at 07385 008242.

☐ I completed the consent form to take part in this study.

Label: Participant ID

Please enter your **Participant ID**

(The one you received from the previous survey)

Notification Initiated Session
[Block 1] Introduction – with graphic (56 prompt)

> Tell me about your most significant **Online** social interaction in the last hour. This can be any social interaction, big or small, positive or negative.
> Tell me about your most significant **In-person** social interaction in the last hour. This can be any social interaction, big or small, positive or negative.

[Block 2] Part 1: Interaction (56 prompts)

> Have you had any ONLINE social interaction in the last hour?
- Yes (Branch: 'Online')
- No, I didn't have online social interaction.
- No, but I had in-person social interaction. (Branch: 'In-person')

> Have you had any In-person social interaction in the last hour?
- Yes (Branch: 'In-person')
- No, I didn't have in-person social interaction
- No, but I had online social interaction. (Branch: 'Online')

Branch: 'Online':
Social platform (1 items – Multiple Choice):

> Please choose the App the interaction took place on...
- WhatsApp / SMS / Emails
- TikTok
- Snapchat
- YouTube

For peer review only

- Instagram
- Twitter (i.e., X)
- BeReal
- Discord
- Facebook
- Roblox
- Pinterest
- Amino
- Reddit
- Others (please specific)

Partner (1 items – Multiple Choice):

> Who was the main person this interaction was with?

- Friends
- Other Peers
- Family
- Known adults
- Someone not known to you
- Others (please specific)

Feeling (2 items – Multiple Choice)

> In relation to the person/people you had the interaction with... To what extent did you feel:

- Strongly rejected
- Rejected
- Neither rejected nor accepted
- Accepted
- Strongly accepted
- Not applicable

> In relations to the person/people you had the interaction with... To what extent did you feel:

- Strongly unpleasant
- Unpleasant
- Neither unpleasant nor pleasant
- Pleasant
- Strongly pleasant
- Not applicable

Branch: 'In-person':

Partner (1 items – Multiple Choice):

> Who was the main person this interaction was with?

- Friends
- Other Peers
- Family
- Known adults
- Someone not known to you
- Others (please specific)

For peer review only

Feeling (2 items – Multiple Choice)

> In relation to the person/people you had the interaction with…
> To what extent did you feel:
- Strongly rejected
- Rejected
- Neither rejected nor accepted
- Accepted
- Strongly accepted
- Not applicable

> In relations to the person/people you had the interaction with…
> To what extent did you feel:
- Strongly unpleasant
- Unpleasant
- Neither unpleasant nor pleasant
- Pleasant
- Strongly pleasant
- Not applicable

[Block 3] Introduction_Positive (~30 random character images)

> In this section, we will ask you questions related to **POSITIVE** emotions. Tell us, in the last hour, did you feel…

[Block 4] Positive Emotions (5 items – Multiple Choice)

> Happy
- Not at all
- Somewhat
- Moderately
- Very
- Extremely
- Not applicable

> Excited
- Not at all
- Somewhat
- Moderately
- Very
- Extremely
- Not applicable

> Enthusiastic
- Not at all
- Somewhat
- Moderately
- Very
- Extremely
- Not applicable

> Sense of belonging

For peer review only

- Not at all
- Somewhat
- Moderately
- Very
- Extremely
- Not applicable

> Determined
- Not at all
- Somewhat
- Moderately
- Very
- Extremely
- Not applicable

[Block 5] Introduction_Negative (~30 random character images)

> In this section, we will ask you questions related to **NEGATIVE** emotions. Tell us, in the last hour, did you feel…

[Block 6] Negative Emotions (5 items – Multiple Choice)

> Ashamed
- Not at all
- Somewhat
- Moderately
- Very
- Extremely
- Not applicable

> Nervous
- Not at all
- Somewhat
- Moderately
- Very
- Extremely
- Not applicable

> Distressed
- Not at all
- Somewhat
- Moderately
- Very
- Extremely
- Not applicable

> Upset
- Not at all
- Somewhat
- Moderately
- Very
- Extremely
- Not applicable

For peer review only

> Teased
  - Not at all
  - Somewhat
  - Moderately
  - Very
  - Extremely
  - Not applicable

[Block 7] Introduction_Loneliness (~30 random character images)

> In this section, we will ask you questions related to **LONELINESS**. Tell us, in the last hour, did you feel…

[Block 8] Loneliness (3 items – Multiple Choice)

> Lack of companionship
  - Strongly agree
  - Agree
  - Neither agree nor disagree
  - Strongly disagree
  - Disagree
  - Not applicable

> Left out
  - Strongly agree
  - Agree
  - Neither agree nor disagree
  - Strongly disagree
  - Disagree
  - Not applicable

> Isolated
  - Strongly agree
  - Agree
  - Neither agree nor disagree
  - Strongly disagree
  - Disagree
  - Not applicable

[Block 9] Anxiety (2 items – Multiple Choice)

> How anxious did you feel in the last hour?
  - Not at all
  - Somewhat
  - Moderately
  - Very
  - Extremely
  - Not applicable

> How worried did you feel in the last hour?
  - Not at all
  - Somewhat
  - Moderately
  - Very

For peer review only

- Extremely
- Not applicable

[Block 10] Depression (2 items – Multiple Choice)

> How low or depressed did you feel in the last hour?

- Not at all
- Somewhat
- Moderately
- Very
- Extremely
- Not applicable

> How hopeless did you feel in the last hour?

- Not at all
- Somewhat
- Moderately
- Very
- Extremely
- Not applicable

[Block 11] Unpleasant/Pleasant Events (1 item – Multiple Choice)

> In the last hour has anything happened that has affected your emotions positively or negatively that is not linked to social interaction (e.g., want to cinema, exam, missed the bus)

- Yes – Positive (Branch: 'Yes')
- Yes – Negative
- No

Branch Yes:

> How have these events affected your emotions?

- Not at all
- Somewhat
- Moderately
- Very
- Extremely
- Not applicable

[Block 12] Fun Stuff (~54 random images)

[Block 13] Reminder

For peer review only
