# Supplementary material for: Loneliness in the Digital World: protocol for a co-produced ecological momentary assessment study in adolescents
Source: BMJ Open. 2024 Jun 6;14(6):e087374. doi: 10.1136/bmjopen-2024-087374 (PMC11163606; doi:10.1136/bmjopen-2024-087374)
Supplement: Reviewer comments [file bmjopen-2024-087374.reviewer_comments.pdf]

PEER REVIEW HISTORY

BMJ Open publishes all reviews undertaken for accepted manuscripts. Reviewers are asked to complete a checklist review form (**http://bmjopen.bmj.com/site/about/resources/checklist.pdf**) and are provided with free text boxes to elaborate on their assessment. These free text comments are reproduced below.

ARTICLE DETAILS

**TITLE (PROVISIONAL)**	Loneliness in the Digital World: protocol for a co-produced Ecological Momentary Assessment study in adolescents
**AUTHORS**	Xiao, Zhuoni; Robertson, Sarah; Long, Emily; Flaig, Robin; Kirby, Liz; Romaniuk, Liana; Murray, Aja; Whalley, Heather

This article was not externally reviewed at BMJ Open. Protocols that have been independently assessed prior to submission to BMJ Open are usually fast-tracked to publication on the grounds that further substantial changes will not be possible. This independent assessment will usually be external, independent review for both a substantial grant award from a non-commercial or government funder and ethics approval.
